# Jejucarbosides B–E, Chlorinated Cycloaromatized Enediynes, from a Marine *Streptomyces* sp.

**DOI:** 10.3390/md21070405

**Published:** 2023-07-18

**Authors:** Ji Hyeon Im, Yern-Hyerk Shin, Eun Seo Bae, Sang Kook Lee, Dong-Chan Oh

**Affiliations:** Natural Products Research Institute, College of Pharmacy, Seoul National University, Seoul 08826, Republic of Korea; 29cinderella@snu.ac.kr (J.H.I.); yern-hyerk_shin@hms.harvard.edu (Y.-H.S.); ddol1289@snu.ac.kr (E.S.B.); sklee61@snu.ac.kr (S.K.L.)

**Keywords:** enediyne, marine actinomycete, cycloaromatization, cyclopenta[*a*]indene, jejucarboside

## Abstract

Four new chlorinated cycloaromatized enediyne compounds, jejucarbosides B–E (**1**–**4**), were discovered together with previously-identified jejucarboside A from a marine actinomycete strain. Compounds **1**–**4** were identified as new chlorinated cyclopenta[*a*]indene glycosides based on 1D and 2D nuclear magnetic resonance, high-resolution mass spectrometry, and circular dichroism (CD) spectra. Jejucarbosides B and E bear a carbonate functional group whereas jejucarbosides C and D are variants possessing 1,2-diol by losing the carbonate functionality. It is proposed that the production of **1**–**4** occurs via Bergman cycloaromatization capturing Cl^-^ and H^+^ in the alternative positions of a *p*-benzyne intermediate derived from a 9-membered enediyne core. Jejucarboside E (**4**) displayed significant cytotoxicity against human cancer cell lines including SNU-638, SK-HEP-1, A549, HCT116, and MDA-MB-231, with IC_50_ values of 0.31, 0.40, 0.25, 0.29, and 0.48 μM, respectively, while jejucarbosides B–D (**1**–**3**) showed moderate or no cytotoxic effects.

## 1. Introduction

Enediynes are a class of highly reactive and potent organic compounds that contain a unique structural feature known as an enediyne core [1]. Enediynes have gained significant attention in the field of medicinal chemistry due to their potent cytotoxic activity and unique DNA cleavage mechanism. DNA cleavage occurs through a process called Bergman cycloaromatization [2], where the enediyne forms a highly reactive diradical species that attacks the DNA molecule, leading to its fragmentation and cell death.

Enediynes are classified into two groups: 9-membered and 10-membered enediyne, depending on the ring sizes of the enediyne core [3]. Their core structures comprise a double bond or double bond equivalent flanked by two triple bonds. One well-known example of a 9-membered enediyne compound is neocarzinostatin [4], which was the first enediyne discovered from *Streptomyces carzinostaticus*. Neocarzinostatin is an extremely potent anticancer agent and has shown promising results in the treatment of solid tumors including liver carcinoma. It has been developed as an anticancer drug (SMANCS) [5], demonstrating the pharmaceutical potential of 9-membered enediynes. Since the initial discovery of neocarzinostatin, 9-memebered enediynes have been considered as a captivating group of organic compounds. They are known for their potent cytotoxic properties and have consistently attracted significant interest in anticancer drug research. However, 9-membered enediynes are more unstable than 10-membered enediynes, and uncyclized 9-membered enediynes are mostly discovered as chromoprotein complexes, requiring complicated isolation schemes [6] encompassing both protein and small molecule purification steps. Considering that 9-membered enediynes are extremely rare, i.e., only five intact uncyclized 9-membered enediynes (neocarzinostatin [1], C-1027 [7], kedaricidin [8], maduropeptin [9], and N1999A2 [10]) and four cycloaromatized 9-membered enediynes (sporolide [11], cyanosporaside [12], fijiolide [13], and amycolamycin [14]) have been reported over the approximately 40 years since neocarzinostatin was discovered in 1985. Therefore, the efficient selection of strains which can express 9-membered enediyne biosynthetic gene clusters [15,16], and thus produce enediyne compounds, is required rather than relying on conventional random search strategies.

**Figure 1:**
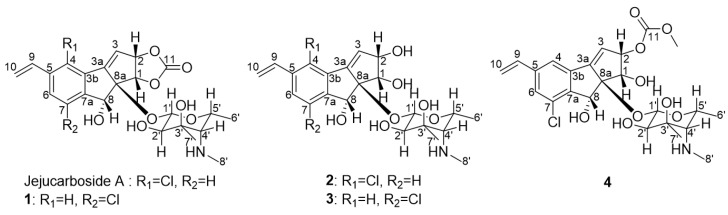
Structures of jejucarbosides B–E (**1**–**4**), along with jejucarboside A.

Our recent genomic and spectroscopic signature-based discovery of 9-membered enediyne-derived cycloaromatized compounds involved the screening of a bacterial DNA library (1080 strains), from which we selected 62 actinobacterial strains bearing 9-membered enediyne core genes [17]. The subsequent cultivation of the strains in Cl-enriched media and the identification of Cl-bearing compounds in their extracts based on the characteristic isotopic signature of Cl successfully identified the first 9-membered enediyne product, jejucarboside A, since the discovery of amycolamycins A and B [14] in 2017. The discovery of structurally novel jejucarboside A bearing a new amino sugar, i.e., 3-methyl-4-methylamino-4,6-dideoxy-d-gulose, coupled with enediyne core-derived chlorinated cyclopenta[*a*]indene aglycone incorporating carbonate functionality may lead to subsequent studies seeking to diversify the structures of 9-membered enediyne-derived natural products. Since the discovery of jejucarboside A from marine *Streptomyces* sp. JJC13, collected on Jeju Island, Republic of Korea, extensive subculture selection was performed, because its yield was extremely low, i.e., 8 μg/L. The production level was subsequently enhanced by approximately 150 folds, resulting in a final yield of 1.25 mg/L, which enabled further chemical exploration, allowing us to discover new jejucarboside A congeners and thereby broadening the chemical diversity of 9-membered enediyne-derived compounds. In this study, we report the discovery, structure elucidation, and biological activity of four new chlorinated cycloaromatized enediyne-derived compounds, jejucarbosides B–E (**1**–**4**) (Figure 1).

## 2. Results and Discussion

### 2.1. Discovery and Structure Elucidation

Chemical analysis of the strain *Streptomyces* sp. JJC13 identified a series of compounds, along with the previously-reported jejucarboside A [15]. The UV spectra of the compounds were virtually identical to that of jejucarboside A, and their mass spectrometric data clearly displayed the characteristic mono-chlorinated pattern, [M+H]^+^:[M+2+H]^+^ = 3:1 (Figure S1). Therefore, these compounds were initially identified as jejucarboside A congeners, and thus, enediyne-derived cycloaromatized compounds, prompting us to perform large scale cultivation, chromatographic purification, and structure elucidation.

Jejucarboside B (**1**) was isolated as a white powder, and its molecular formula was deduced as C_23_H_26_ClNO_8_ based on its high-resolution electrospray ionization mass spectrometric (HRESIMS) data with ^1^H and ^13^C nuclear magnetic resonance (NMR) spectra (Figures S2 and S3). Further analysis of the ^1^H and ^13^C NMR data (Table 1) indicated that this compound is structurally related to jejucarboside A (Figure 1). However, two downfield singlet ^1^H signals (*δ*_H_ 7.68 and 7.49) were clearly different from the two doublet protons (*δ*_H_ 7.73 and 7.46) in jejucarboside A; this observation led us to perform a comprehensive structural analysis. An analysis of the ^1^H, ^13^C, and HSQC NMR spectra revealed all ^1^H-^13^C one-bond correlations. Four sp^2^ methine protons (*δ*_H_ 7.68, 7.49, 6.76, and 6.30), one olefinic methylene (*δ*_H_ 5.92 and 5.40), one dioxygenated methine proton (*δ*_H_ 4.38), six heteroatom-bearing methine protons (*δ*_H_ 5.57, 5.34, 5.21, 3.97, 2.99, and 1.88), and three methyl groups (*δ*_H_ 2.40, 1.19, and 1.10) were identified. The ^13^C NMR data showed that **1** showed polyunsaturated and polyoxygenated features with eleven carbons in the double-bond/carbonyl regions (*δ*_C_ 156.5–117.4), nine carbons in the oxygenated chemical shift region (*δ*_C_ 99.7–68.8), and the last three carbons in the aliphatic area (*δ*_C_ 39.1, 23.6, and 16.6).

The planar structure of jejucarboside B (**1**) was determined by analyzing the COSY and HMBC correlations. First of all, two singlet protons (H-4 and H-6 at *δ*_H_ 7.68 and 7.49, respectively) were coupled in the COSY spectrum, indicating that they were located in *meta*-positions in a six-membered aromatic ring. H-4 displayed HMBC correlations with C-6 and C-7a, while H-6 coupled with C-4 and C-7a, with C-7a being assigned to a benzene ring. Based on the HMBC correlations from H-9 to C-4, C-5, and C-6, C-5 was assigned between C-4 and C-6. ^2^*J*_H4C3b_ and ^2^*J*_H6C7_ HMBC correlations revealed the positions of C-3b and C-7 in the six-membered aromatic ring. The H-9/H_2_-10 COSY and H_2_-10/C-5 HMBC correlations indicated the presence of olefinic methylene carbon in C-10, adjacent to C-9.

COSY correlations of H-2 to H-1 and H-3, along with HMBC coupling from H-1 to C-8a and C-3a and from H-3 to C-3a and C-8a, revealed a cyclopentene moiety. The H-1/C-11 and H-2/C-11 HMBC signals indicated that the carbonate carbon C-11 formed a 1,3-dioxolane ring with oxygen atoms at C-1 and C-2. The HMBC correlations of singlet methine proton H-8 to C-3a, C-3b, C-7a, and C-8a suggested a cyclopenta[*a*]indene structure.

**Figure 2:**
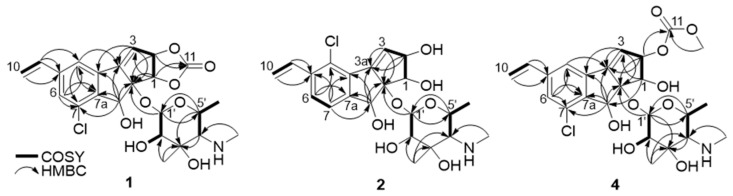
Key COSY and HMBC correlations of jejucarbosides B (**1**), C (**2**), and E (**4**).

The COSY signal from H-1′ to H-2′ confirmed that the dioxygenated carbon C-1′ and oxygenated carbon C-2′ were connected. H_3_-7′, one of the singlet methyl groups, exhibited HMBC correlations with C-2′, C-3′, and C-4′, thus assigning C-7′ to the fully substituted carbon, C-3′. The other singlet methyl group, H_3_-8′, had HMBC correlation with C-4′, which revealed that C-8′ was connected to C-4′ via a nitrogen atom. The chemical shift of C-8′ was 39.1 ppm, which suggested the existence of a methylamino group, rather than a methoxy group. Based on the H_3_-6′/H-5′ COSY correlation, the doublet methyl group at C-6′ was connected to C-5′. The COSY correlation between H-4′ and H-5′ secured the C-4′-C-5′ connectivity. The HMBC signals from H-1′ to C-5′ and H-5′ to C-1′ indicated the presence of an amino sugar as a hexose structure. The sugar moiety was then connected to C-8a in the cyclopenta[*a*]indene aglycone via H-1′/C-8 HMBC correlation. After elucidating most of the structure of **1**, one chlorine atom remained, and C-7 still required a functional group to complete the structure. The chlorine atom in the molecular formula was determined to be located at C-7, differing from jejucarboside A, which bears a chlorine atom at C-4 (Figure 2)

**Figure 3:**
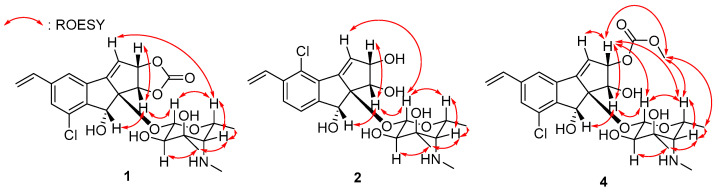
Key ROESY correlations of jejucarbosides B (**1**), C (**2**), and E (**4**).

ROESY correlations and homonuclear and heteronuclear coupling constants allowed us to determine the relative configuration of jejucarboside B (**1**). Based on the ^1^*J*_C1′H1′_ value (160 Hz), we established the presence of a *β*-configuration and thus assigned H-1′ to an axial position [18]. A large diaxial coupling constant was measured between H-1′ and H-2′ (7.5 Hz), indicating H-2′ as the axial position. A ROESY signal from H-1′ to H-5′ allowed us to assign H-5′ to an axial position on the same phase as H-1′. H_3_-7′ correlated with the axial proton H-2′ in the ROESY spectrum, locating the C-7′ methyl group to an equatorial position. The coupling constant between H-4′ and H-5′ was nearly 0 Hz, indicating an equatorial position for H-4′. H-4′/H-5′ and H-4′/H_3_-6′ ROESY correlations also supported the equatorial assignment of H-4′. Therefore, the hexose was determined to be 3-methyl-4-methylamino-4,6-dideoxy-d-gulose (Figure 3).

**Figure 4:**
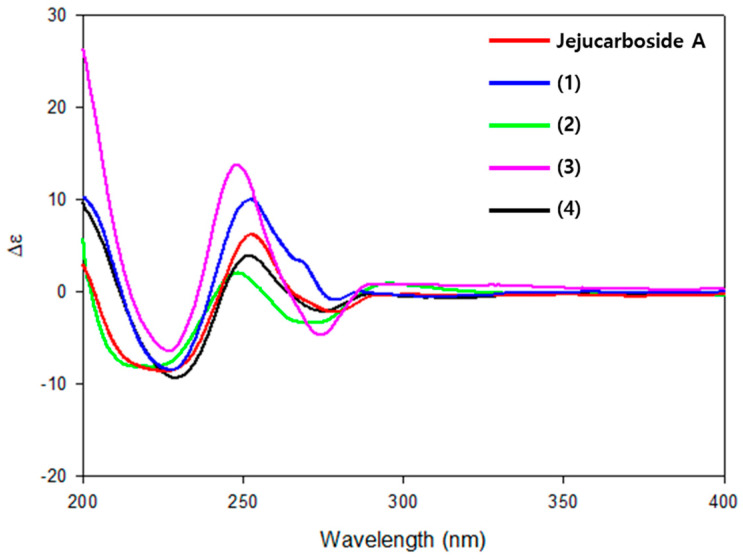
Experimental CD spectrum of jejucarbosides A–E.

In the aglycone, H-1 exhibited ROESY signals with H-2 and H-8, suggesting that they were oriented to the same phase. Furthermore, H-1/H-1′ and H-3/H-5′ ROESY correlation revealed the relative configuration of jejucarboside B (**1**) as 1S*, 2S*, 8R*, 8aR*, 1′S*, 2′R*, 3′R*, 4′S*, and 5′R*, which is identical to that of jejucarboside A (Figure 3). The absolute configuration of **1** was deemed to be the same as that of jejucarboside A by comparing the experimental circular dichroism (CD) spectra of the two compounds. They commonly showed a distinct negative Cotton effect at 227 nm and a positive effect at 254 nm in their CD spectra, revealing the absolute configuration of **1** as 1*S*, 2*S*, 8*R*, 8a*R*, 1′*S*, 2′*R*, 3′*R*, 4′*S*, and 5′*R*, as determined previously for jejucarboside A (Figure 4).

Jejucarboside C (**2**) was purified as a white powder. Based on HRESIMS and NMR data, its molecular formula was determined to be C_22_H_28_ClNO_7_, with 10 double bond equivalents. The ^1^H NMR spectrum of **2** displayed the same number of protons as jejucarboside A, and their splitting patterns were identical to those in jejucarboside A. Analysis of the ^1^H, ^13^C, and HSQC NMR data indicated ^1^H-^13^C one bond correlations and revealed that H-1, H-2, C-1, and C-2 were more shielded than those in jejucarboside A: H-1 and H-2 in jejucarboside A (*δ*_H_ 5.32 and 5.59) were shifted to upfield in **2** (*δ*_H_ 4.25 and 4.59), while C-1 (*δ*_C_ 70.5) and C-2 (*δ*_C_ 79.8) of **2** were also detected in the upper field, as opposed to the corresponding carbons (*δ*_C_ 74.4 and 87.7) in jejucarboside A, indicating the modification of the dioxolane ring. In addition, the carbonyl carbon at 156.6 ppm in jejucarboside A disappeared in **2**. Therefore, the structure of jejucarboside C (**2**) was determined to possess a 1,2-diol moiety at C-1 and C-2 and a chlorine atom at C-4 by losing the carbonate carbon from jejucarboside A (Figure 2). It was established that the configuration of **2** was identical to those of jejucarbosides A and B by analysis of ROESY correlations and ^1^H-^1^H coupling constants, followed by circular dichroism (CD) spectral analysis (Figure 3 and Figure 4).

Jejucarboside D (**3**) was isolated as a white powder. The molecular formula of **3** was determined to be C_22_H_28_ClNO_7_, which is identical to that of **2**, based on its HRESIMS data. The ^1^H NMR spectra of **3** exhibited the same splitting pattern as **1**, but the chemical shifts of H-1 and H-2 were shifted to the upper field, which indicated that the relationship between **1** and **3** is analogous to that between jejucarboside A and **2**. A detailed analysis of the 1D and 2D NMR spectra assigned all the ^1^H and ^13^C atoms and thus to identify **3** as a new analogue of **1** without a carbonate carbonyl group (Figure 1). The absolute configuration of **3** was deduced to be identical to that of **1** by comparing their electronic CD spectra (Figure 4).

Jejucarboside E (**4**) was also yielded as a white powder by chromatographic purification. The HRESIMS data of **4** revealed its molecular formula to be C_24_H_30_ClNO_9_, which bears additional CH_4_O. Comparing the ^1^H NMR spectrum of **4** with those of **1**–**3** indicated that jejucarboside E (**4**) is structurally analogous to **1**–**3**; however, this comparison also clearly identified an additional methoxy group (*δ*_H_ 3.77). Our analysis of the 1D and 2D NMR spectra assigned all the protons and carbons. The methoxy protons displayed a HMBC correlation to C-11, i.e., the carbonate carbon. In addition, C-1/H-1 were observed at *δ*_C_ 68.9/*δ*_H_ 4.54, indicating that C-**1** contained a hydroxy group, like jejucarbosides C and D. Unlike **1**, only H-2 showed a HMBC correlation to C-11, and the chemical shifts of C-2 and H-2 were 84.0 and 5.61 ppm, indicating that the carbonate functional group was connected to C-2. Therefore, jejucarboside E (**4**) was determined to be a new congener of **1** bearing a methoxy group connected to the carbonate group at C-2. An analysis of the ROESY spectrum supported the position of the methoxy group by H_3_-11-OMe/H-2 correlation and established the relative configuration (Figure 3). The absolute configuration of **4** was deemed to be identical to those of **1**–**3** based on their consistent ECD spectra (Figure 4).

### 2.2. Proposed Mechanism of Production of Jejucarbosides B–E

**Figure 5:**
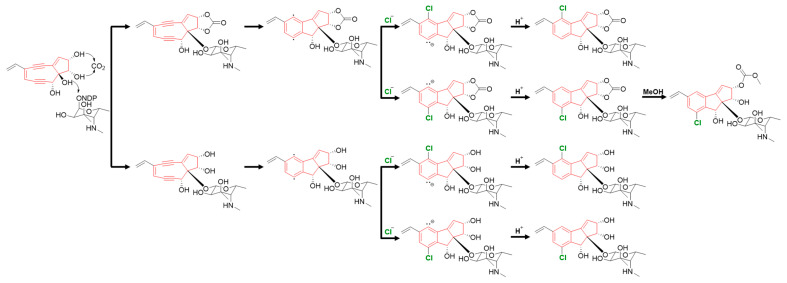
Proposed mechanism of the production of jejucarbosides B–E.

Jejucarbosides B–E (**1**–**4**) are new congeners of jejucarboside A. A genomic analysis of the jejucarboside producer strain, *Streptomyces* sp. JJC13, revealed only one enediyne biosynthetic gene cluster (BGC), leading us to propose the putative enediyne biosynthetic pathway for jejucarboside A. Jejucarboside B (**1**) has chlorine at C-7, whereas C-4 is chlorinated in jejucarboside A. This variation can be explained by the biradical formation of a *p*-benzyne intermediate during the Bergman cycloaromatization of the 9-membered enediyne core [1]. Cl^-^ can be incorporated at either radical position of C-4 and C-7. Once chloride forms a bond with a radical-bearing carbon, the radical in the other carbon is converted to a haloaryl anion, and the anion is trapped by H^+^ to form mono-chlorinated cyclopenta[*a*]indene structures (Figure 5).

Therefore, theoretically, both jejucarbosides A and B should be produced at a 1:1 ratio. In the time course analysis of the metabolic profiles of JJC13, these compounds were produced at a 1:1 ratio, even though the yield of **1** was lower than that of jejucarboside A after purification, because **1** required more complicated chromatographic procedures. However, not every cycloaromatized enediyne generate both chlorinated compounds at a 1:1 ratio. C-1027 chromophore-V and fijiolides have only one chlorinated form, probably because of steric hindrance due to their more complicated ring structures. Jejucarbosides C and D (**2**–**3**) are new compounds without carbonate functionality from the same enediyne pathway as jejucarbosides A and B. Jejucarboside D (**4**) could be produced by methoxylation at the carbonate carbon from **1** (Figure 5).

### 2.3. Biological Activity Jejucarbosides B–E

The cytotoxicity of jejucarbosides B–E (**1**–**4**) was evaluated against the five human cancer cell lines, i.e., HCT116 (colon cancer cells), A549 (lung cancer cells), SNU-638 (gastric carcinoma), SK-HEP-1 (hepatic carcinoma), and MDA-MB-231 (breast cancer cells). Jejucarbosides B–C (**1**–**2**) showed moderate cytotoxic effects, and jejucarboside D (**3**) exhibited no notable inhibitory activity. However, jejucarboside E (**4**) showed significant cytotoxic activity against all the tested cancer cell lines, with IC_50_ values of 0.31, 0.40, 0.25, 0.29, and 0.48 μM for the SNU-638, SK-HEP-1, A549, HCT116, and MDA-MB-231 cell lines, respectively (Table 2). This result indicates that the structural moiety, in combination with carbonate and methoxy functional groups, contributes to increasing cytotoxicity against the tested cancer cell lines.

Based on the structures of the jejucarbosides, the chlorination positions (C-4 or C-7) did not significantly affect the cytotoxic activity against the tested cancer cells. The existence of a 1,3-dioxolan-2-one structure with carbonate functionality did not result in a significant difference in activity. However, it was clearly shown that the structural moiety composed of carbonate and methoxy functional groups in jejucarboside E (**4**) significantly increased the cytotoxicity against the tested cancer cell lines.

## 3. Materials and Methods

### 3.1. General Experiment Procedures

Optical rotations were measured using a JASCO P-2000 polarimeter (sodium light source, JASCO, Easton, PA, USA) with a 1-cm cell at 25 °C. Ultraviolet (UV) and circular dichroism (CD) spectra were acquired using a Chiralscan plus Applied Photophysics spectrophotometer (Applied Photophysics, Leatherhead, Surrey, UK). Infrared (IR) spectral data were recorded by using a JASCO Fourier transform/infrared spectrometer (FT/IR-4200, Tokyo, Japan). ^1^H, ^13^C, and 2D NMR spectra were acquired on a Bruker Avance 850 MHz spectrometer (Bruker, Billerica, MA, USA) at the National Center for Inter-University Research Facilities (NCIRF) at Seoul National University. Low-resolution electrospray ionization mass spectrometry (LR-ESI-MS) and UV chromatogram data were acquired using an Agilent Technologies 6130 quadrupole mass spectrometer (Agilent Technologies, Santa Clara, CA, USA) coupled with an Agilent Technologies 1200-series HPLC using a reversed-phase C_18_(2) column (Phenomenex Luna, 5 μm, 100 × 4.6 mm). High-resolution electrospray ionization mass spectrometry (HR-ESI-MS) data were acquired using an AB Sciex TripleTOF 5600 HR-MS spectrometer (Q-TOF 5600, Framingham, MA, USA) at the National Instrumentation Center for Environment Management (NICEM) in the College of Agriculture and Life Sciences, Seoul National University.

### 3.2. Bacterial Strain and Subculture Selection

Strain JJC13 was isolated from a marine sediment sample collected at Hyeopjae Beach, Jeju Island, Republic of Korea, in May 2020, as reported previously. The JJC13 strain was identified as a *Streptomyces* sp. that was closely related to *Streptomyces sanglieri* (99% similarity, GenBank accession No. AB735535) [17,20] on the basis of the 16S rDNA sequence (GenBank accession No.ON428251). Initially, the yield of jejucarboside A was extremely low (only ~ 8 μg/L). To enhance the yield of the jejucarbosides, successive subculture selection and culture optimization were performed. Selecting subcultures and monitoring jejucarboside production by LC/MS enabled to increase the titer to 1.25 mg/L, which is ~150 fold better than that of the initial culture. For the optimization of the culture conditions to produce the jejucarbosides, the cultures of *Streptomyces* sp. JJC13 in 10 different media (YEME, A1C, YEME+humic acid, YEME-l-ornithine, YEME+humic acid+l-ornithine, DSY, A1, GLY, modified K, and YPM; for compositions, see Table S2) were monitored. Among the 10 different media, YEME (4 g of yeast extract, 4 g of glucose, 10 g of malt extract, 14 g of sea salt in 1 L of distilled water) provided the highest yield of jejucarbosides. A subsequent comparative study using baffled and round flasks was performed, during which we monitored the production of the jejucarbosides in culture extracts by LC/MS analysis. When the bacteria were cultivated in baffled flasks, the yield of the jejucarbosides was higher than in normal Erlenmeyer flasks. Consequently, for large-scale culture, 2.8-L Ultra-yield flasks with baffles were utilized. In the time course culture analysis, the cultivation time was optimized to 5 days for the large-scale culture.

### 3.3. Scale-up Culture and Extraction

Frozen stock of *Streptomyces* sp. JJC13 was inoculated in a 125 mL Erlenmeyer flask containing 50 mL of YEME liquid medium (4 g of yeast extract, 4 g of glucose, 10 g of malt extract, and 14 g of sea salt in 1 L of distilled water) and cultured for 3 days on a rotary shaker (180 rpm, 30 °C). Then, 10 mL of the culture was inoculated in a 500 mL Erlenmeyer flask containing 250 mL YEME liquid medium supplemented with sea salt. After 3 days of cultivation, 10 mL of the culture was transferred to a 2.8 L Ultra-yield flask containing 1 L of YEME liquid medium with sea salt for scale-up. In total, 10 L of liquid cultivation was used for five more days under the same conditions. All the cultivates (10 L) were extracted with EtOAc using a separation funnel. After separating the EtOAc and water layers, anhydrous sodium sulfate was added to the EtOAc layer to remove residual water. The EtOAc extract was concentrated *in vacuo* to dry material using a rotary evaporator.

### 3.4. Isolation of Jejucarbosides B–E (***1***–***4***)

The dried bacterial extract was dissolved in MeOH, adsorbed on Celite, and the mixture was dried in vacuo. The adsorbed Celite mixture was loaded onto a C_18_ reversed-phase open column (YMC C_18_ resin, 60 × 40 mm). The extract was fractionated by eluting a step gradient composed of water and MeOH (20, 40, 60, 80, and 100% MeOH-H_2_O, 300 mL each). Aliquots (20 µL) of the fractions were analyzed using LC/MS under a gradient system, which indicated that jejucarbosides B–E were eluted in the 20% and 40% MeOH-H_2_O fractions. The dried 20% and 40% MeOH-H_2_O fractions were dissolved in MeOH and filtered through a hydrophilic syringe filter (ADVANTEC, 25HP045AN) to remove insoluble small particles. Jejucarbosides B–E were further purified using semi-preparative reversed-phased HPLC (reversed-phase C_18_ YMC^®^ column, 5 μm, 250 × 10 mm) using a gradient solvent system (10%–30% CH_3_CN-H_2_O over 30 min, flow rate: 2 mL/min, detection: UV 254 nm). Jejucarbosides B–E were eluted at 25–36 min after injection, and then further purification was conducted under isocratic HPLC conditions (10% CH_3_CN-H_2_O, flow rate: 0.6 mL/min, detection: UV 254 nm) using a chiral HPLC column (CHIRALPAK IB, 250 × 4.6 mm, 5 μm). In the final purification, jejucarboside B (**1**) (2.0 mg), C (**2**) (2.5 mg), D (**3**) (2.7 mg), and E (**4**) (1.9 mg) were eluted at retention times of 14, 11, 10 and 13 min, respectively.

Jejucarboside B (**1**): white powder, ${\left[\alpha \right]}_{D}^{20}$ = −41.8 (c 0.1, MeOH); UV (MeOH) λ_max_ (log ε) 247 (3.75) nm; ECD (c 2.0 × 10^−4^ M, MeOH) λ_max_ (Δε) 227 (−12.15), 254 (13.83); IR (neat) ν_max_ 3402, 1786, 1593, and 1049 cm^−1^; for ^1^H and ^13^C NMR data, see Table 1, HR-ESI-MS m/z 480.1427 [M+H]^+^ (calcd for C_23_H_27_ClNO_8_H, 480.1419).

Jejucarboside C (**2**): white powder, ${\left[\alpha \right]}_{D}^{20}$ = −11.5 (c 0.1, MeOH); UV (MeOH) λ_max_ (log ε) 247 (3.75) nm; ECD (c 2.0 × 10^−4^ M, MeOH) λ_max_ (Δε) 221 (−11.19), 248 (2.77); IR (neat) ν_max_ 3359, 1595, and 1073 cm^−1^; for ^1^H and ^13^C NMR data, see Table 1, HR-ESI-MS m/z 454.1626 [M+H]^+^ (calcd for C_23_H_27_ClNO_8_H, 454.1627).

Jejucarboside D (**3**): white powder, ${\left[\alpha \right]}_{D}^{20}$ = −15.0 (c 0.1, MeOH); UV (MeOH) λ_max_ (log ε) 247 (3.75) nm; ECD (c 2.0 × 10^−4^ M, MeOH) λ_max_ (Δε) 227 (−8.82), 248 (18.89); IR (neat) ν_max_ 3390, 1599, and 1046 cm^−1^; for ^1^H and ^13^C NMR data, see Table 1, HR-ESI-MS m/z 454.1627 [M+H]^+^ (calcd for C_23_H_27_ClNO_8_H, 454.1627).

Jejucarboside E (**4**): white powder, ${\left[\alpha \right]}_{D}^{20}$ = −15.9 (*c* 0.1, MeOH); UV (MeOH) λ_max_ (log ε) 247 (3.75) nm; ECD (c 2.0 × 10^−4^ M, MeOH) λ_max_ (Δε) 229 (−14.48), 252 (4.97); IR (neat) *ν*_max_ 3395, 1783, 1594, and 1072 cm^−1^; for ^1^H and ^13^C NMR data, see Table 1, HR-ESI-MS *m*/*z* 512.1681 [M+H]^+^ (calcd for C_23_H_27_ClNO_8_H, 512.1681).

### 3.5. Cytotoxicity Assay

The cytotoxicity of jejucarbosides B–E was evaluated using a sulforhodamine B (SRB) assay, as previously reported [21]. Five human cancer cell lines, A549 (Human lung cancer cells), MDA-MB-231 (Human breast cancer cells), HCT116 (Human colon cancer cells), SNU-638 (Human gastric carcinoma), and SK-Hep-1 (Human hepatic carcinoma), were tested. The A549, MDA-MB-231, HCT116, SK-Hep-1 cancer cell lines were obtained from the American Type Culture Collection (Manassas, VA, USA). The SNU-638 cell line was provided by the Korean Cell Line Bank (Seoul, Korea). Cells were cultivated in medium (RPMI-1640 media for A549, HCT116, and SNU-638 cells; DMEM media for MDA-MB-231 and SK-Hep-1 cells) containing 10 % fetal bovine serum (FBS) and 1% of penicillin-streptomycin solution. Cells were seeded in 96-well plates and incubated for 30 min for zero-day controls or treated with test compounds for 72 h. After incubation, the cells were fixed with 10% TCA for 30 min, dried, and stained with 0.4% SRB solution in 1% acetic acid for 2 h. The stained cells were suspended in 10 mM Tris (pH 10.0). The optical density was measured at 515 nm and the survival rates were determined. The growth inhibitory concentration (IC_50_) was calculated via non-linear regression analysis using TableCurve 2D v5.01 software (Systant Software Inc., Richmond, CA, USA).

## 4. Conclusions

Chemical analysis of jejucarboside A-producing marine *Streptomyces* strain resulted in discovering new congeners, i.e., jejucarbosides B–E (**1**–**4**). These compounds commonly bear chlorinated cyclopenta[*a*]indene glycoside structures. We propose that they were derived from a 9-membered enediyne precursor that was biosynthesized through an enediyne biosynthetic pathway via coupling with a unique sugar, 3-methyl-4-methylamino-4,6-dideoxy-d-gulose, and cycloaromatization. The alternative positions of Cl in the jejucarbosides originated from a *p*-benzyne intermediate capturing Cl^-^ and subsequently H^+^. In our biological evaluation, the jejucarbosides were not expected to be cytotoxic because they are cycloaromatized compounds, rather than intact enediynes. However, jejucarboside E (**4**) displayed submicromolar cytotoxicity against the tested cancer cell lines (SNU-638, SK-HEP-1, A549, HCT116, and MDA-MB-231), indicating that the structural moiety containing carbonate and methoxy functional groups may increase the cytotoxicity in this enediyne-derived chlorinated cyclopenta[*a*]indene glycoside family. Considering that 9-membered enediyne-derived natural products are extremely rare, with less than 10 families [1], the discovery of compounds **1**–**4** contributes significantly to our knowledge of 9-membered enediyne compounds. Marine actinomycetes may therefore be valuable sources of rare enediynes.

## Figures and Tables

**Table 1:** NMR spectroscopic data of jejucarbosides B–E (**1**–**4**) (850 MHz, CD_3_CN).

	Jejucarboside B (1)		Jejucarboside C (2)		Jejucarboside D (3)		Jejucarboside E (4)	
Position	*δ*_C_, Type	*δ*_H_, Mult (*J* in Hz)	*δ*_C_, Type	*δ*_H_, Mult (*J* in Hz)	*δ*_C_, Type	*δ*_H_, Mult (*J* in Hz)	*δ*_C_, Type	*δ*_H_, Mult (*J* in Hz)
1	74.6, CH	5.34, d (7.0)	70.5, CH	4.25, d (5.5)	70.7, CH	4.28, d (5.0)	68.9, CH	4.54, d (6.0)
2	87.5, CH	5.57, dd (7.0, 3.0)	79.8, CH	4.59, dd (5.5, 3.0)	79.6, CH	4.58, dd (5.0, 3.0)	84.0, CH	5.61, dd (6.0, 3.0)
3	122.4, CH	6.30, d (3.0)	129.9, CH	6.57. d (3.0)	127.6, CH	6.34, d (3.0)	123.0, CH	6.27, d (3.0)
3a	156.5, C		150.3, C		152.0, C		154.6, C	
3b	137.7, C		134.7, C		138.4, C		137.8, C	
4	120.3, CH	7.68, s	129.6, C		120.1, CH	7.58, s	120.5, CH	7.60, s
5	142.1, C		137.5, C		141.8, C		141.8, C	
6	129.2, CH	7.49, s	128.8, CH	7.63, d (8.0)	128.3, CH	7.42, s	128.8, CH	7.44, s
7	133.2, C		126.5, CH	7.40, d (8.0)	133.5, C		133.5, C	
7a	146.6, C		152.6, C		147.4, C		147.6, C	
8	73.3, CH	5.21, s	73.8, CH	5.01, s	72.5, CH	5.19, s	72.7, CH	5.13, s
8a	95.1, C		96.6, C		96.2, C		96.4, C	
9	135.9, CH	6.76, dd (18.0, 11.0)	133.2, CH	7.13, dd (17.5, 11.0)	136.2, CH	6.75, dd (18.0, 11.0)	136.1, CH	6.73, dd (17.5, 11.0)
10	117.4, CH_2_	5.40, d (11.0)	118.3, CH_2_	5.46, d (11.0)	116.9, CH_2_	5.36, d (11.0)	117.1, CH_2_	5.36, d (11.0)
		5.92, d (18.0)		5.85, d (17.5)		5.88, d (18.0)		5.88, d (17.5)
11	156.5, C						156.2, C	
11-OMe							55.5, CH_3_	3.77, s
1′	99.7, CH	4.38, d (7.5)	98.6, CH	4.95, d (8.0)	99.3, CH	5.02, d (8.0)	98.3, CH	4.93, d (8.0)
2′	72.8, CH	2.99, d (7.5)	73.3, CH	3.08, d (8.0)	72.8, CH	3.09, d (8.0)	73.1, CH	3.07, d (8.0)
3′	76.0, C		76.1, C		75.9, C		76.1, C	
4′	68.8, CH	1.88, s	68.8, CH	1.95, d (1.5)	68.8, CH	1.97, d (1.5)	68.8, CH	2.00, d (1.5)
5′	70.0, CH	3.97, q (6.5)	70.3, CH	4.20, qd (6.5, 1.5)	70.1, CH	4.24, qd (6.5, 1.5)	70.4, CH	4.32, qd (6.5, 1.5)
6′	16.6, CH_3_	1.10, d (6.5)	17.0, CH_3_	1.13, d (6.5)	16.5, CH_3_	1.15, d (6.5)	17.0, CH_3_	1.17, d (6.5)
7′	23.6, CH_3_	1.19, s	23.6, CH_3_	1.22, s	23.7, CH_3_	1.23, s	23.6, CH_3_	1.23, s
8′	39.1, CH_3_	2.40, s	39.1, CH_3_	2.41, s	39.1, CH_3_	2.41, s	39.1, CH_3_	2.43, s

**Table 2:** Cytotoxic activity of jejucarbosides B–E (**1**–**4**). Etoposide [19] was used as a positive control.

(μM)	SNU638	SK-Hep-1	A549	HCT116	MDA-MB-231
Jejucarboside A	>100	>100	>100	29.30	>100
Jejucarboside B	14.17	41.55	26.09	16.47	42.38
Jejucarboside C	16.40	46.63	25.17	21.33	43.20
Jejucarboside D	>100	>100	>100	>100	>100
Jejucarboside E	0.31	0.40	0.25	0.29	0.48
Etoposide	0.20	0.63	0.21	0.56	4.48

## Data Availability

All data is contained within this article and Supplementary Materials.

## Supplementary Materials

The following supporting information can be downloaded at: https://www.mdpi.com/article/10.3390/md21070405/s1, Figure S1: Figure S1. LC/MS profile of the extract of *Streptomyces* sp. JJC13 showing the characteristic mono-chlorinated pattern ([M+H]^+^:[M+2+H]^+^ = 3:1) of jejucarbosides A-E; Figure S2. ^1^H NMR spectrum of jejucarboside B (**1**) at 850 MHz in CD_3_CN; Figure S3. ^13^C NMR spectrum of jejucarboside B (**1**) at 850 MHz in CD_3_CN; Figure S4. COSY NMR spectrum of jejucarboside B (**1**) at 850 MHz in CD_3_CN; Figure S5. HSQC NMR spectrum of jejucarboside B (**1**) at 850 MHz in CD_3_CN; Figure S6. HMBC NMR spectrum of jejucarboside B (**1**) at 850 MHz in CD_3_CN; Figure S7. ROESY NMR spectrum of jejucarboside B (**1**) at 850 MHz in CD_3_CN; Figure S8. ^1^H NMR spectrum of jejucarboside C (**2**) at 850 MHz in CD_3_CN; Figure S9. ^13^C NMR spectrum of jejucarboside C (**2**) at 850 MHz in CD_3_CN; Figure S10. COSY NMR spectrum of jejucarboside C (**2**) at 850 MHz in CD_3_CN; Figure S11. HSQC NMR spectrum of jejucarboside C (**2**) at 850 MHz in CD_3_CN; Figure S12. HMBC NMR spectrum of jejucarboside C (**2**) at 850 MHz in CD_3_CN; Figure S13. ROESY NMR spectrum of jejucarboside C (**2**) at 850 MHz in CD_3_CN; Figure S14. ^1^H NMR spectrum of jejucarboside D (**3**) at 850 MHz in CD_3_CN; Figure S15. ^13^C NMR spectrum of jejucarboside D (**3**) at 850 MHz in CD_3_CN; Figure S16. COSY NMR spectrum of jejucarboside D (**3**) at 850 MHz in CD_3_CN; Figure S17. HSQC NMR spectrum of jejucarboside D (**3**) at 850 MHz in CD_3_CN; Figure S18. HMBC NMR spectrum of jejucarboside D (**3**) at 850 MHz in CD_3_CN; Figure S19. ROESY NMR spectrum of jejucarboside D (**3**) at 850 MHz in CD_3_CN; Figure S20. ^1^H NMR spectrum of jejucarboside E (**4**) at 850 MHz in CD_3_CN; Figure S21. ^13^C NMR spectrum of jejucarboside E (**4**) at 850 MHz in CD_3_CN; Figure S22. COSY NMR spectrum of jejucarboside E (**4**) at 850 MHz in CD_3_CN; Figure S23. HSQC NMR spectrum of jejucarboside E (**4**) at 850 MHz in CD_3_CN; Figure S24. HMBC NMR spectrum of jejucarboside E (**4**) at 850 MHz in CD_3_CN; Figure S25. ROESY NMR spectrum of jejucarboside E (**4**) at 850 MHz in CD_3_CN; Table S1. NMR spectroscopic data of jejucarboside A (800 MHz, CD_3_CN) and jejucarbosides B-E (**1**-**4**) (850 MHz, CD_3_CN); Table S2. Composition of media used for culture optimization; Phylogenetic analysis of Enediyne Core Gene Amplicons; Figure S26. Phylogenetic tree of the amplicons; References [22,23].
