# Supplementary material for: Jejucarbosides B–E, Chlorinated Cycloaromatized Enediynes, from a Marine Streptomyces sp
Source: Mar Drugs. 2023 Jul 18;21(7):405. doi: 10.3390/md21070405 (PMC10381858; doi:10.3390/md21070405)
Supplement: Supplementary file 1 [file marinedrugs-21-00405-s001.zip › marinedrugs-2499605-Supplementary Materials.pdf]

Supporting Information

Jejucarbosides B-E, Chlorinated Cycloaromatized Enediynes, from a Marine *Streptomyces* sp.

Ji Hyeon Im[1], Yern-Hyerk Shin[1], Eun Seo Bae[1], Sang Kook Lee[1], and Dong-Chan Oh[1,*]

[1]Natural Products Research Institute, College of Pharmacy, Seoul National University, Seoul 08826, Republic of Korea

Correspondence to:
Prof. Dong-Chan Oh
Natural Products Research Institute,
College of Pharmacy,
Seoul National University, 1 Gwanak-ro, Gwanak-gu,
Seoul 08826, Republic of Korea
TEL: 82-2-880-2491; FAX: 82-2-762-8322; E-mail: dongchanoh@snu.ac.kr

Table of Contents

Figure S1. LC/MS profile of the extract of *Streptomyces* sp. JJC13 showing the characteristic mono-chlorinated pattern ($[M+H]^+$:$[M+2+H]^+$ = 3:1) of jejucarbosides A-E. S3
Figure S2. ^{1}H NMR spectrum of jejucarboside B (**1**) at 850 MHz in CD_3CN. S6
Figure S3. ^{13}C NMR spectrum of jejucarboside B (**1**) at 850 MHz in CD_3CN. S6
Figure S4. COSY NMR spectrum of jejucarboside B (**1**) at 850 MHz in CD_3CN. S7
Figure S5. HSQC NMR spectrum of jejucarboside B (**1**) at 850 MHz in CD_3CN. S7
Figure S6. HMBC NMR spectrum of jejucarboside B (**1**) at 850 MHz in CD_3CN. S8
Figure S7. ROESY NMR spectrum of jejucarboside B (**1**) at 850 MHz in CD_3CN. S8
Figure S8. ^{1}H NMR spectrum of jejucarboside C (**2**) at 850 MHz in CD_3CN. S9
Figure S9. ^{13}C NMR spectrum of jejucarboside C (**2**) at 850 MHz in CD_3CN. S9
Figure S10. COSY NMR spectrum of jejucarboside C (**2**) at 850 MHz in CD_3CN. S10
Figure S11. HSQC NMR spectrum of jejucarboside C (**2**) at 850 MHz in CD_3CN. S10
Figure S12. HMBC NMR spectrum of jejucarboside C (**2**) at 850 MHz in CD_3CN. S11
Figure S13. ROESY NMR spectrum of jejucarboside C (**2**) at 850 MHz in CD_3CN. S11
Figure S14. ^{1}H NMR spectrum of jejucarboside D (**3**) at 850 MHz in CD_3CN. S12
Figure S15. ^{13}C NMR spectrum of jejucarboside D (**3**) at 850 MHz in CD_3CN. S12
Figure S16. COSY NMR spectrum of jejucarboside D (**3**) at 850 MHz in CD_3CN. S13
Figure S17. HSQC NMR spectrum of jejucarboside D (**3**) at 850 MHz in CD_3CN. S13
Figure S18. HMBC NMR spectrum of jejucarboside D (**3**) at 850 MHz in CD_3CN. S14
Figure S19. ROESY NMR spectrum of jejucarboside D (**3**) at 850 MHz in CD_3CN. S14
Figure S20. ^{1}H NMR spectrum of jejucarboside E (**4**) at 850 MHz in CD_3CN. S15
Figure S21. ^{13}C NMR spectrum of jejucarboside E (**4**) at 850 MHz in CD_3CN. S15
Figure S22. COSY NMR spectrum of jejucarboside E (**4**) at 850 MHz in CD_3CN. S16
Figure S23. HSQC NMR spectrum of jejucarboside E (**4**) at 850 MHz in CD_3CN. S16
Figure S24. HMBC NMR spectrum of jejucarboside E (**4**) at 850 MHz in CD_3CN. S17
Figure S25. ROESY NMR spectrum of jejucarboside E (**4**) at 850 MHz in CD_3CN. S17
Table S1. NMR spectroscopic data of jejucarboside A (800 MHz, CD_3CN) and jejucarbosides B-E (**1**-**4**) (850 MHz, CD_3CN). S18
Table S2. Composition of media used for culture optimization. S19
Phylogenetic analysis of Enediyne Core Gene Amplicons S20
Figure S26. Phylogenetic tree of the amplicons. S20
References S21

Figure S1. LC/MS profile of the extract of *Streptomyces* sp. JJC13 showing the characteristic mono-chlorinated pattern ($[M+H]^+$:$[M+2+H]^+$ = 3:1) of jejucarbosides A-E.

a. Jejucarboside A

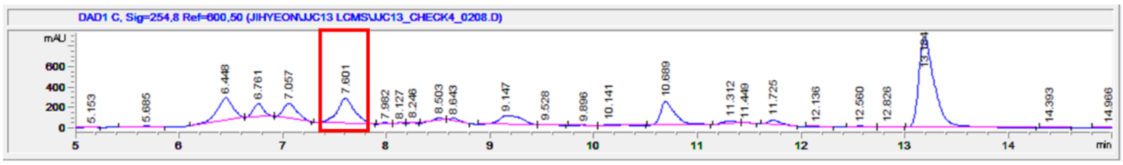

LC/MS chromatogram data (10%−90% CH_3CN-H_2O over 20 min, 0.1% formic acid, flow rate: 0.7 mL/min, detection: UV 254 nm)

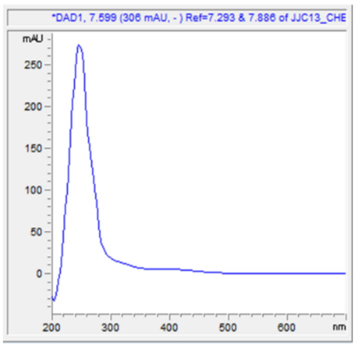

UV Spectrum

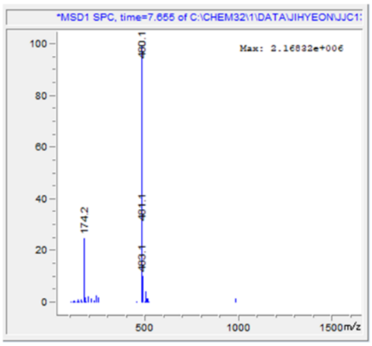

Positive Mass Spectrum

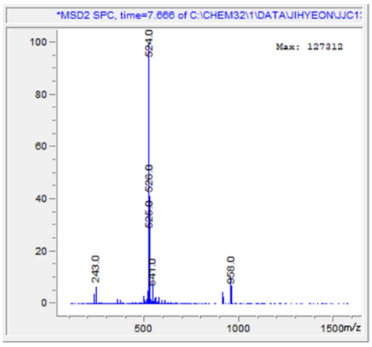

Negative Mass Spectrum

b. Jejucarboside B (**1**)

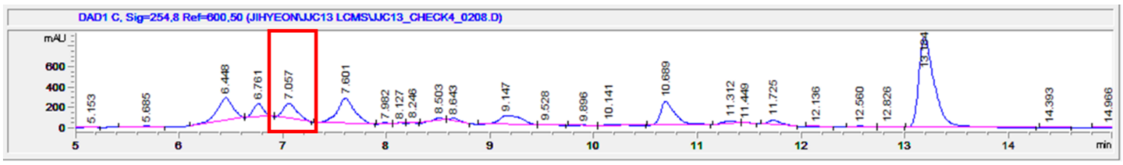

LC/MS chromatogram data (10%−90% CH_3CN-H_2O over 20 min, 0.1% formic acid, flow rate: 0.7 mL/min, detection: UV 254 nm)

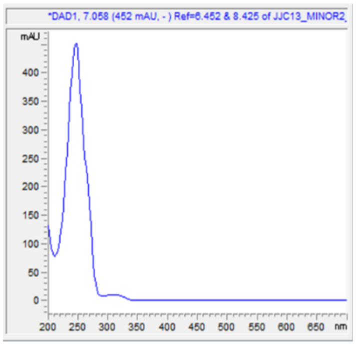

UV Spectrum

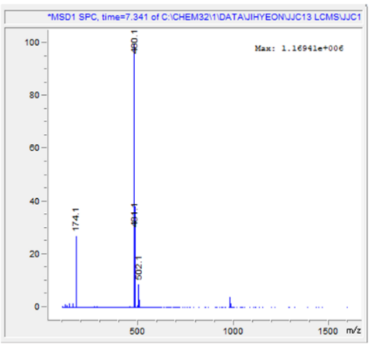

Positive Mass Spectrum

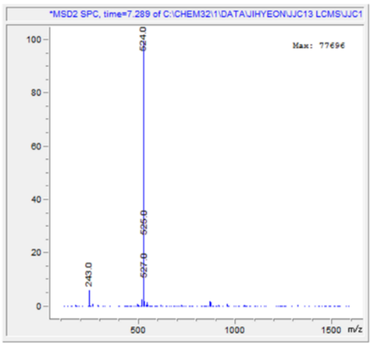

Negative Mass Spectrum

c. Jejucarboside C (**2**)

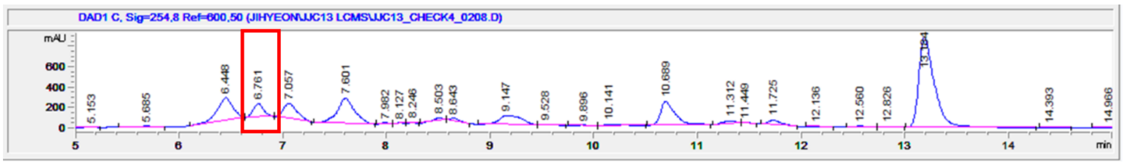

LC/MS chromatogram data (10%−90% CH_3CN-H_2O over 20 min, 0.1% formic acid, flow rate: 0.7 mL/min, detection: UV 254 nm)

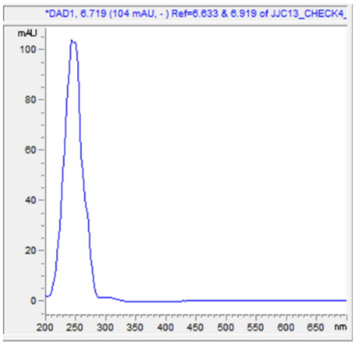

UV Spectrum

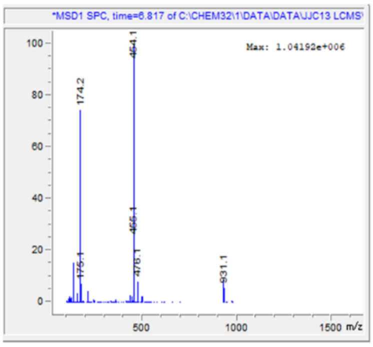

Positive Mass Spectrum

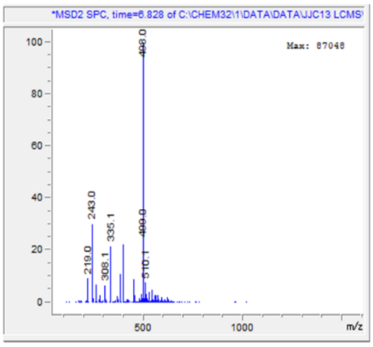

Negative Mass Spectrum

d. Jejucarboside D (**3**)

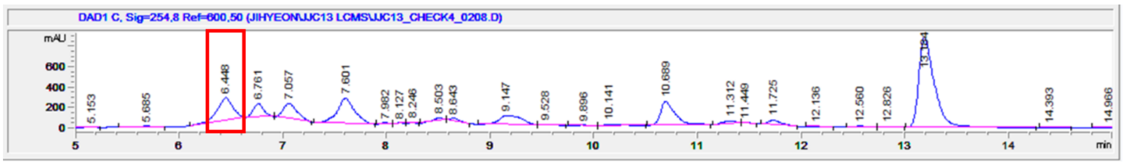

LC/MS chromatogram data (10%−90% CH_3CN-H_2O over 20 min, 0.1% formic acid, flow rate: 0.7 mL/min, detection: UV 254 nm)

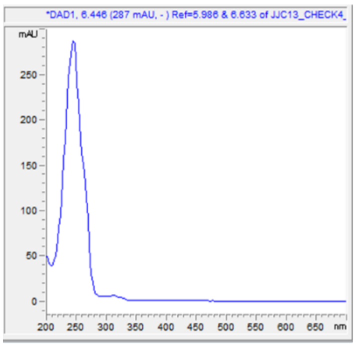

UV Spectrum

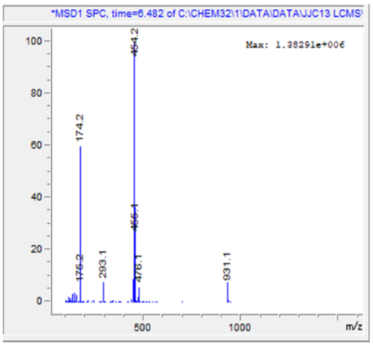

Positive Mass Spectrum

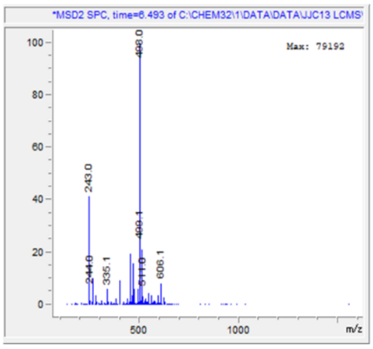

Negative Mass Spectrum

e. Jejucarboside E (**4**)

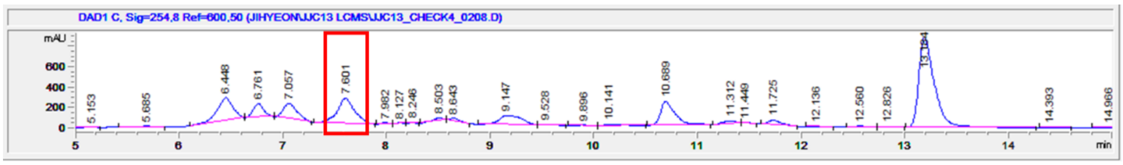

LC/MS chromatogram data (10%–90% CH_3CN–H_2O over 20 min, 0.1% formic acid, flow rate: 0.7 mL/min, detection: UV 254 nm)

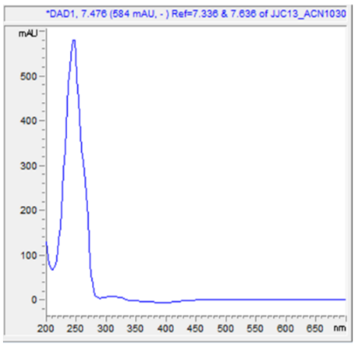

UV Spectrum

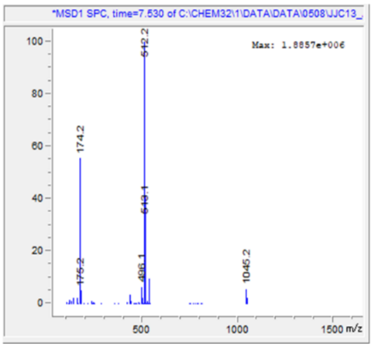

Positive Mass Spectrum

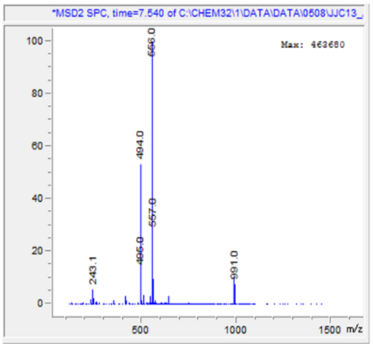

Negative Mass Spectrum

Figure S2. ^{1}H NMR spectrum of jejucarboside B (**1**) at 850 MHz in CD_3CN.

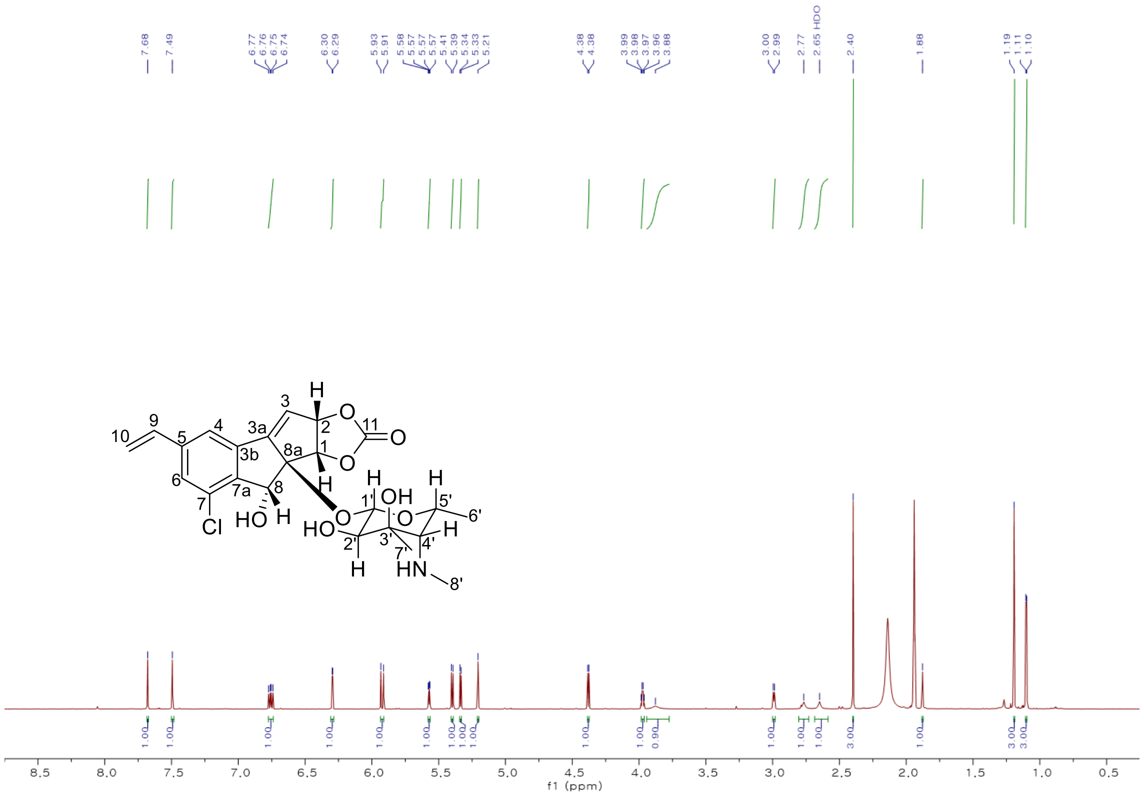

Figure S3. ^{13}C NMR spectrum of jejucarboside B (**1**) at 850 MHz in CD_3CN.

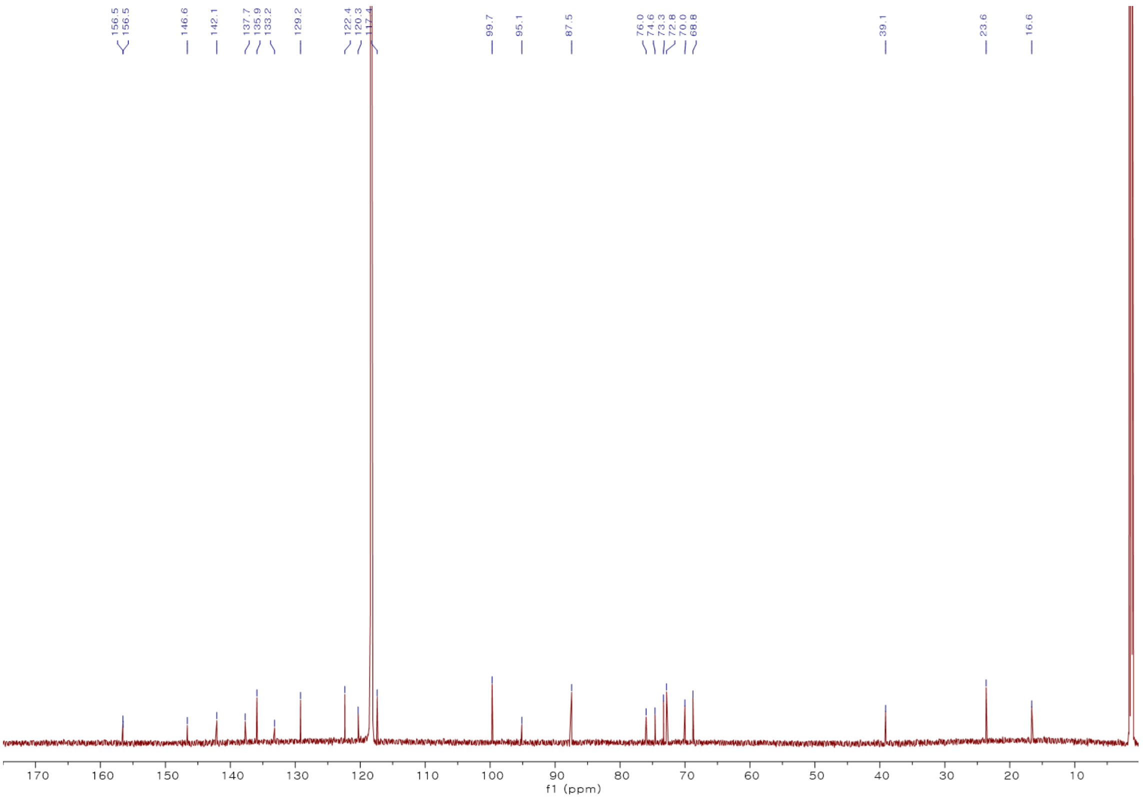

Figure S4. COSY NMR spectrum of jejucarboside B (**1**) at 850 MHz in CD_3CN.

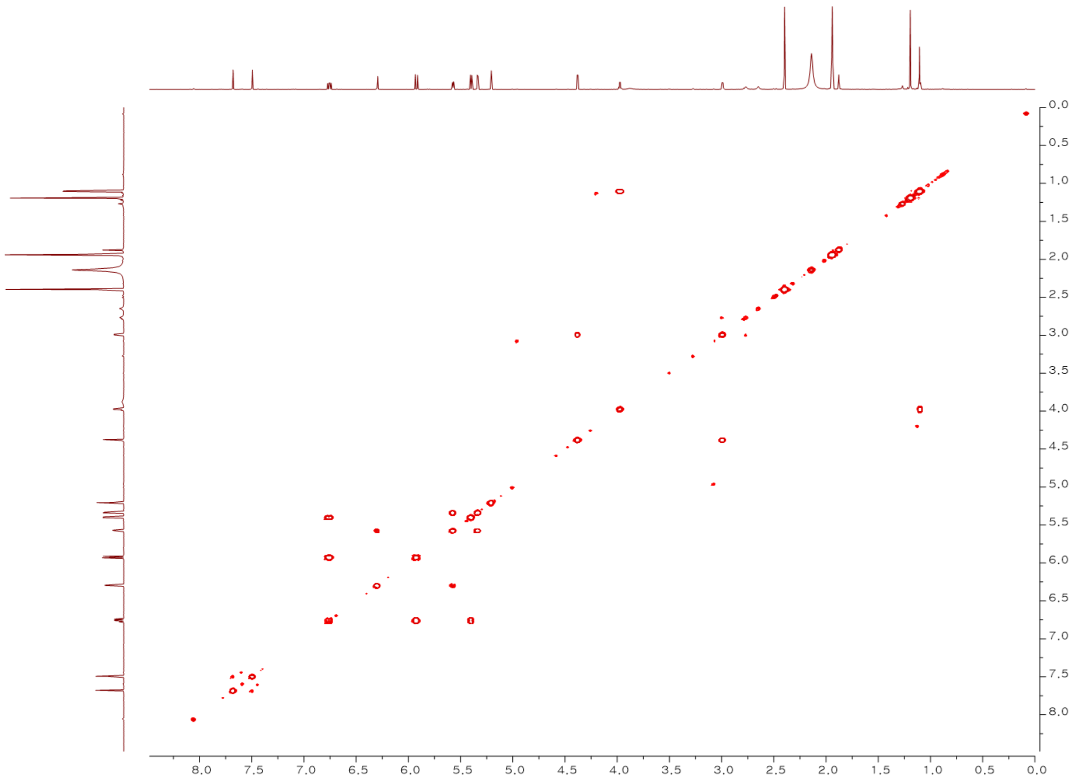

Figure S5. HSQC NMR spectrum of jejucarboside B (**1**) at 850 MHz in CD_3CN.

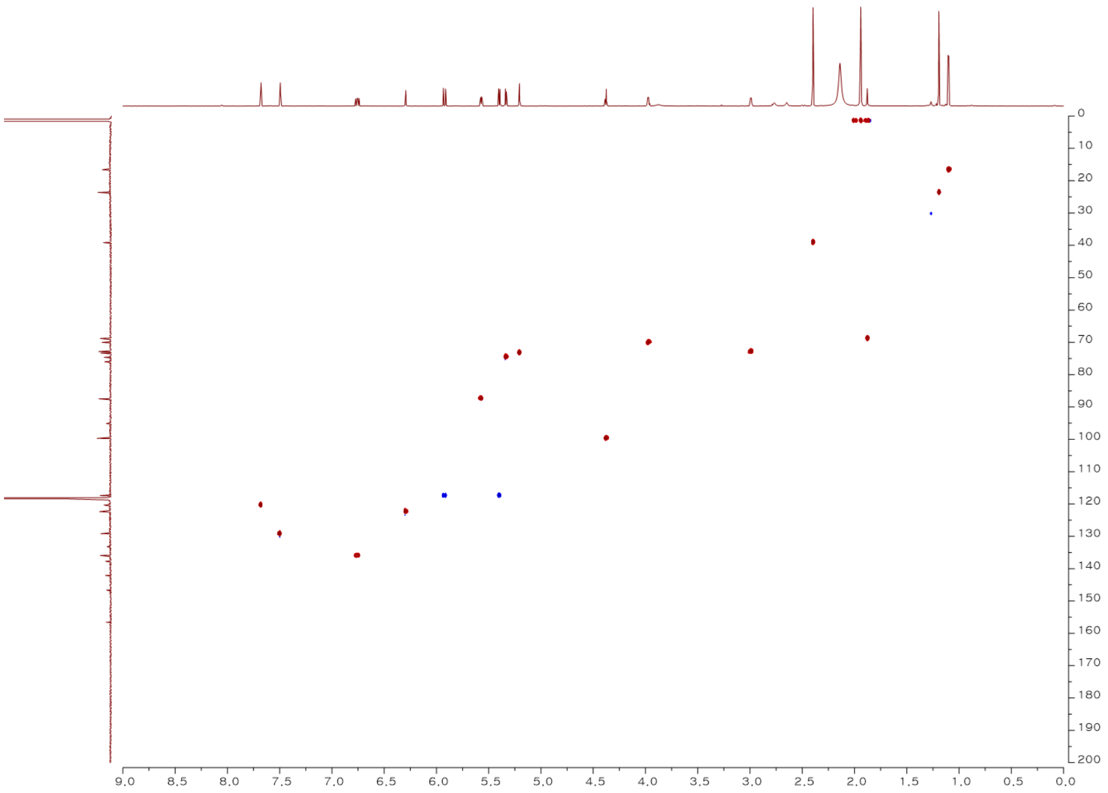

Figure S6. HMBC NMR spectrum of jejucarboside B (**1**) at 850 MHz in CD_3CN.

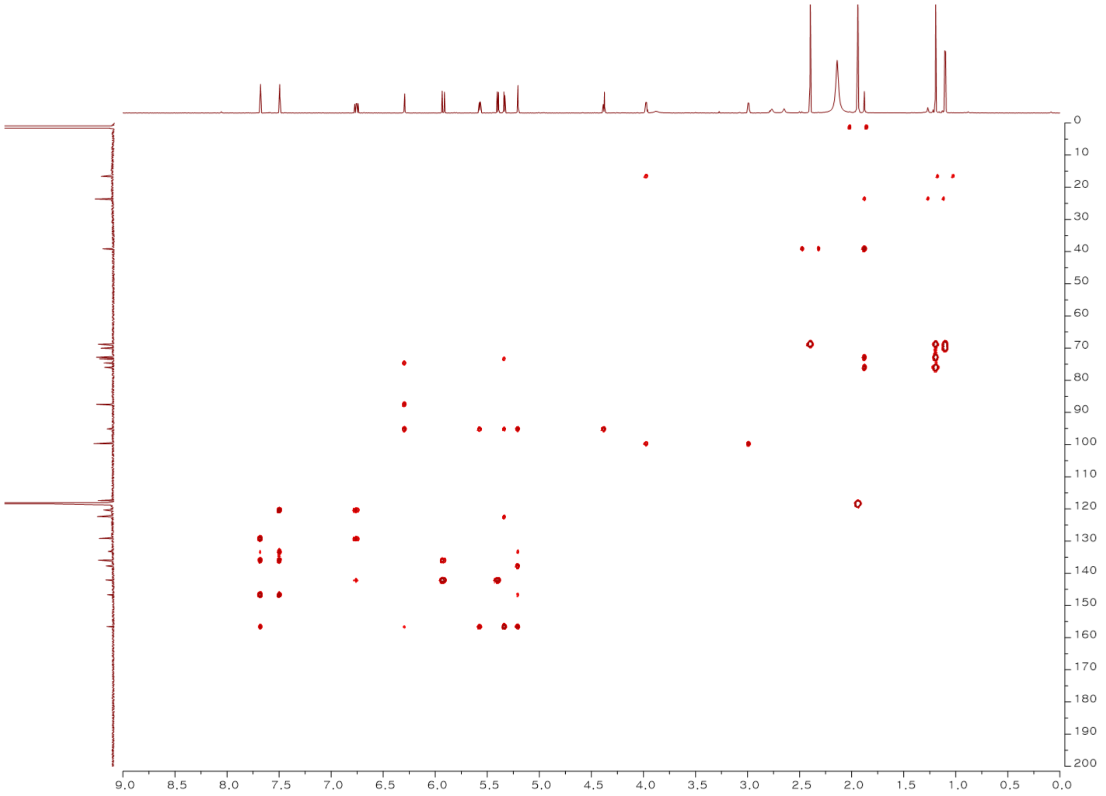

Figure S7. ROESY NMR spectrum of jejucarboside B (**1**) at 850 MHz in CD_3CN.

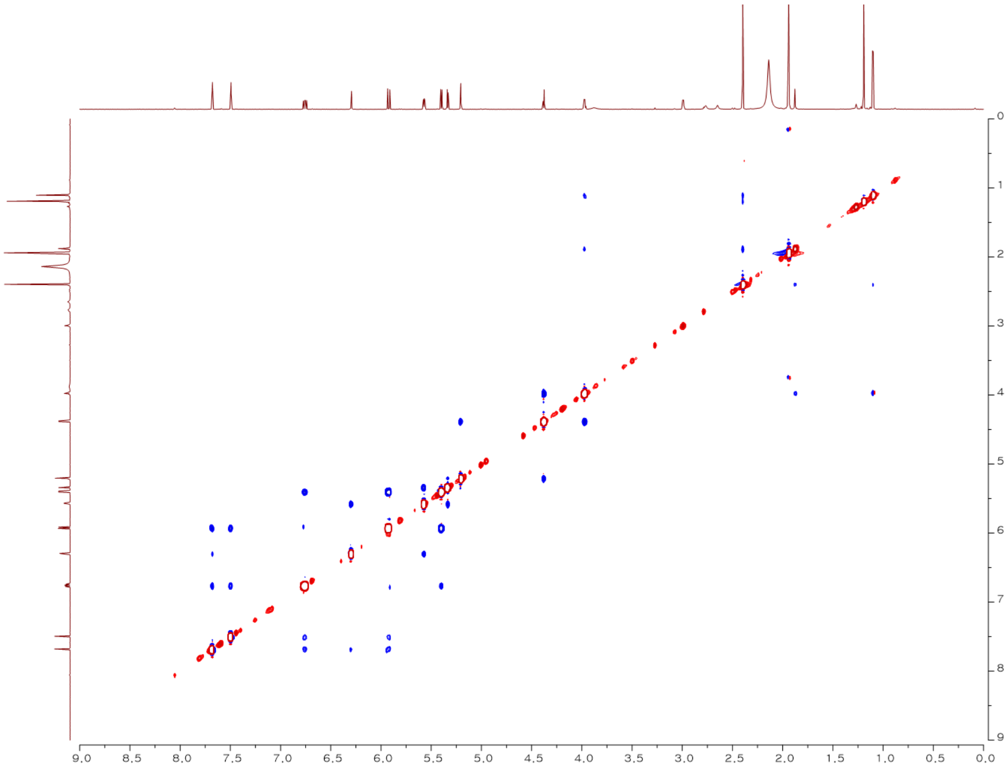

Figure S8. ^{1}H NMR spectrum of jejucarboside C (**2**) at 850 MHz in CD_3CN.

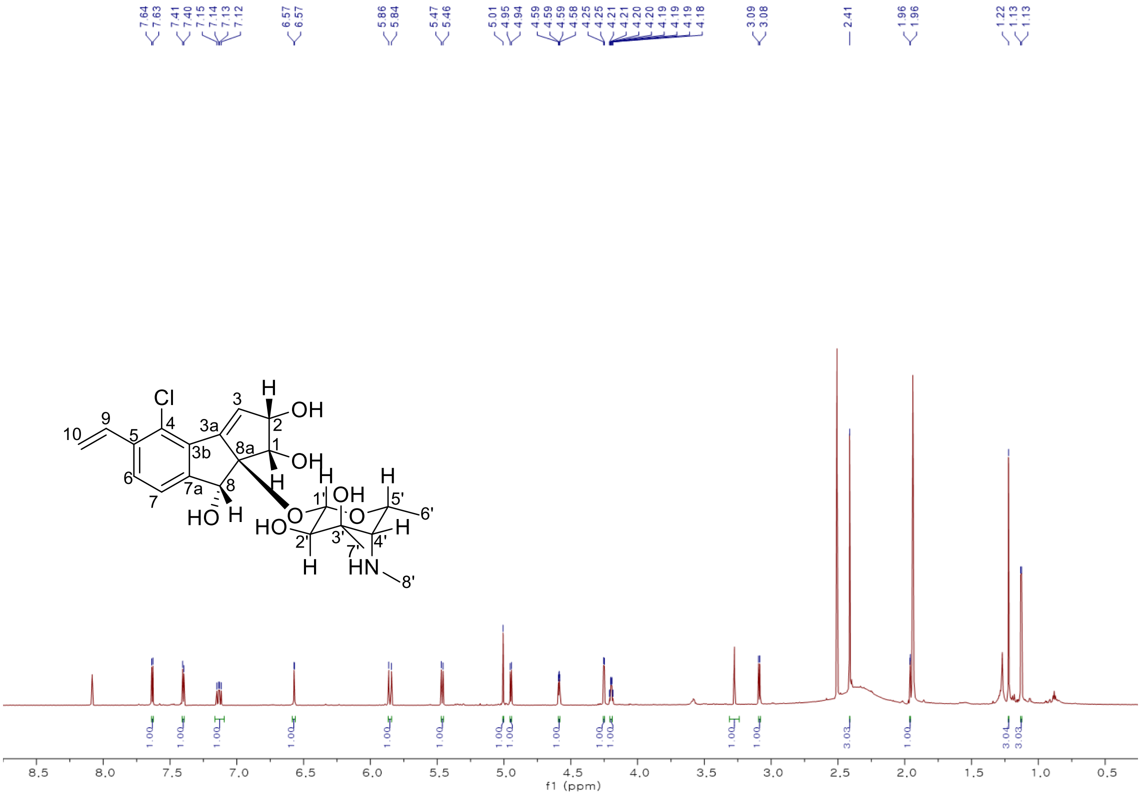

Figure S9. ^{13}C NMR spectrum of jejucarboside C (**2**) at 850 MHz in CD_3CN.

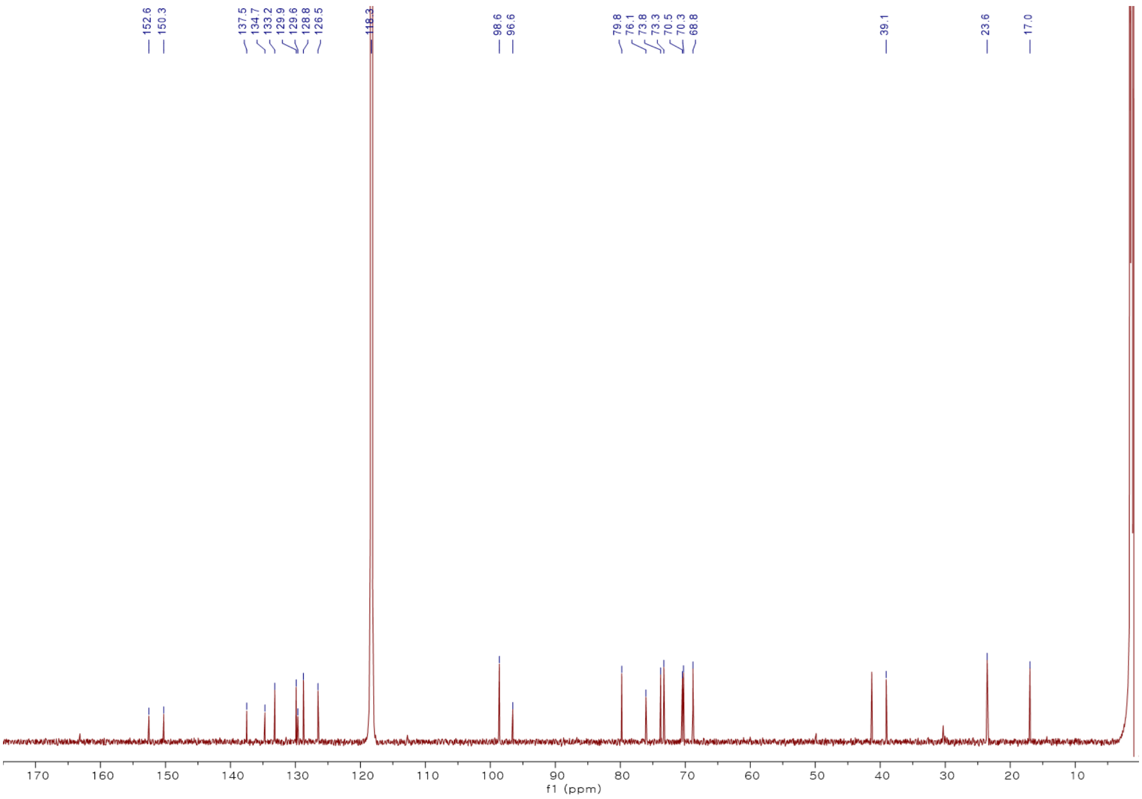

Figure S10. COSY NMR spectrum of jejucarboside C (**2**) at 850 MHz in CD_3CN.

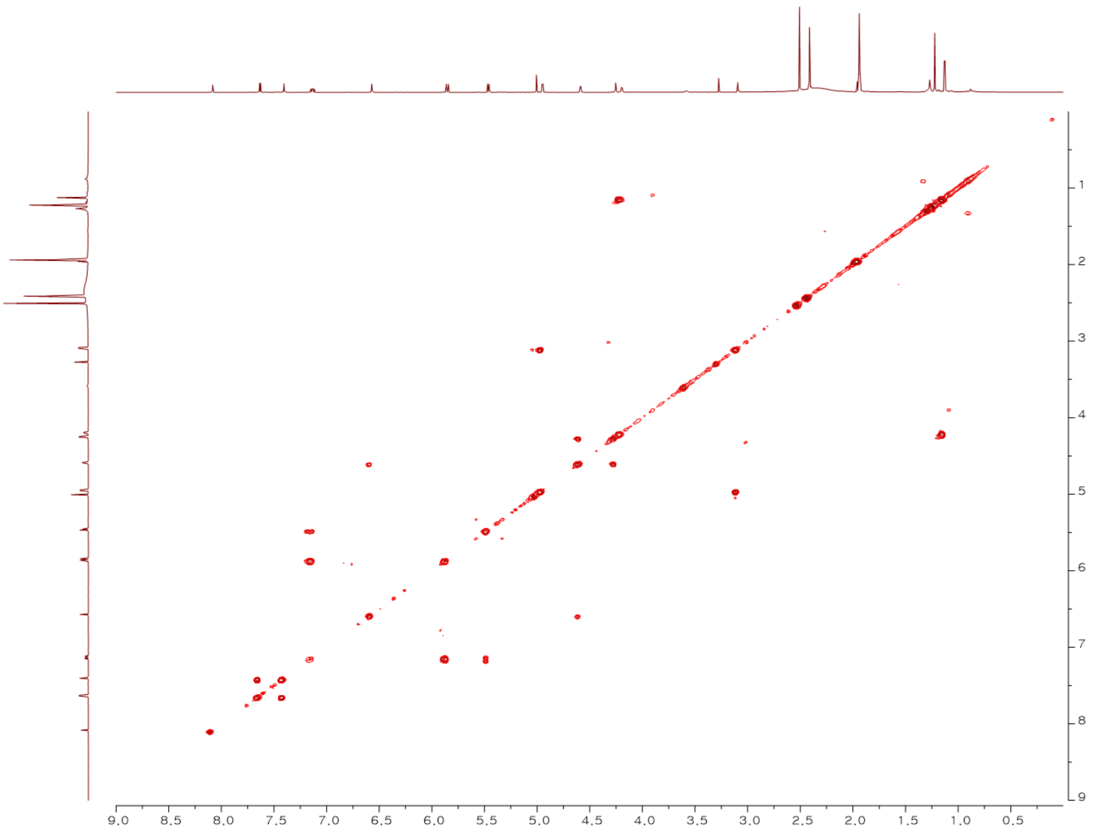

Figure S11. HSQC NMR spectrum of jejucarboside C (**2**) at 850 MHz in CD_3CN.

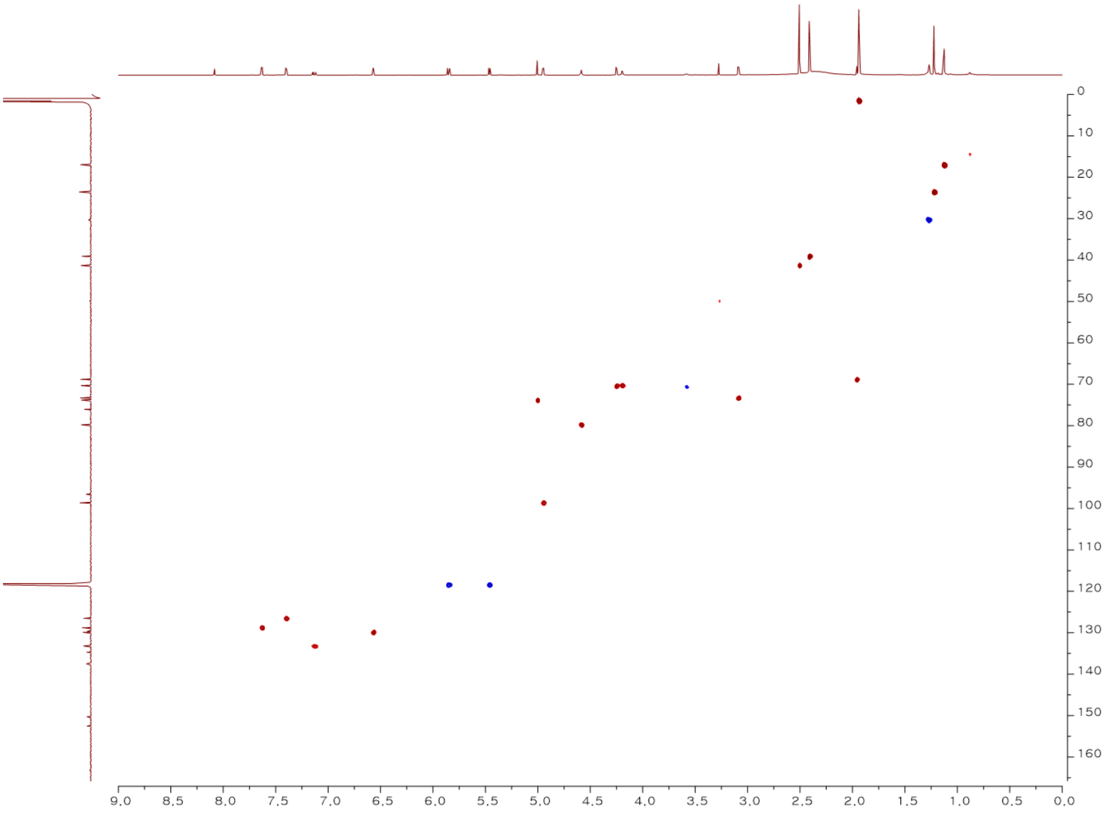

Figure S12. HMBC NMR spectrum of jejucarboside C (**2**) at 850 MHz in CD_3CN.

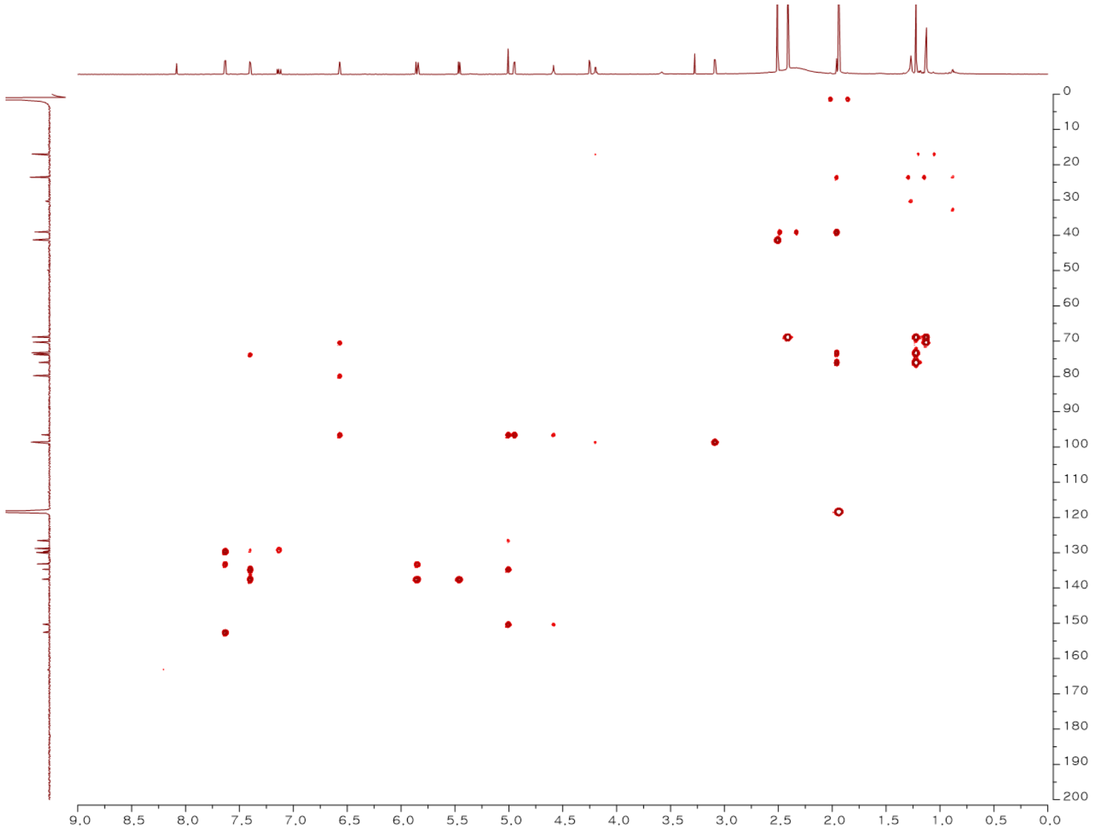

Figure S13. ROESY NMR spectrum of jejucarboside C (**2**) at 850 MHz in CD_3CN.

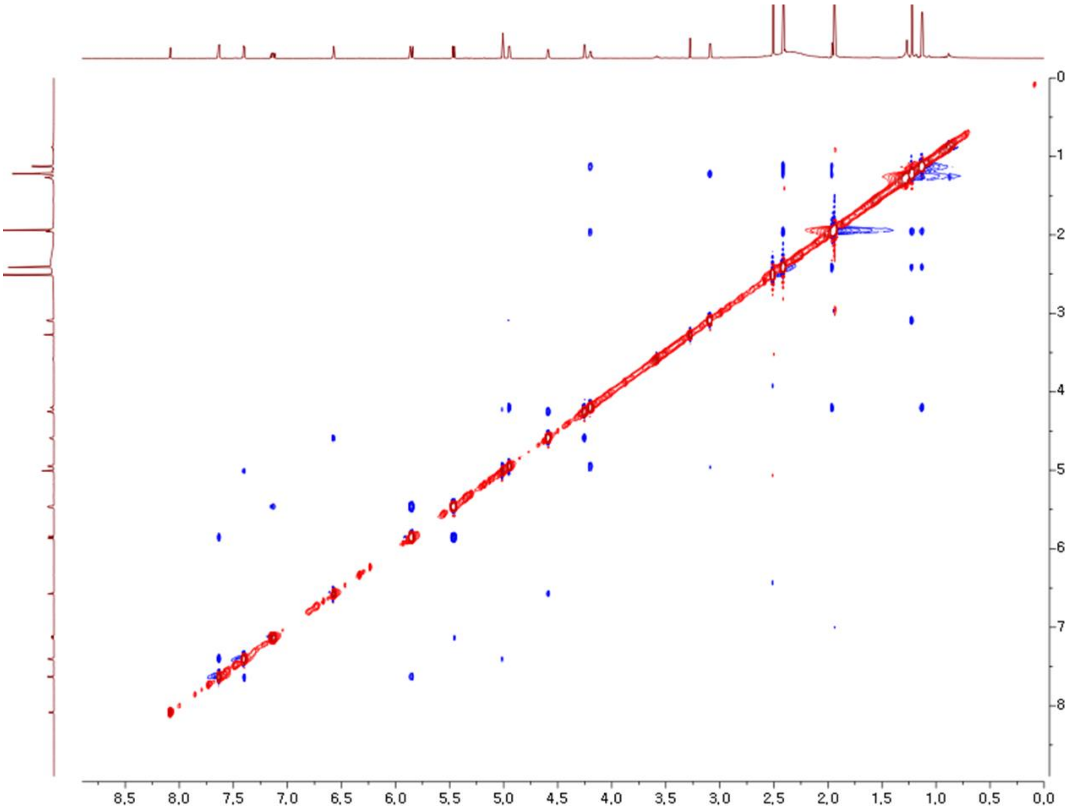

Figure S14. ^{1}H NMR spectrum of jejucarboside D (**3**) at 850 MHz in CD_3CN.

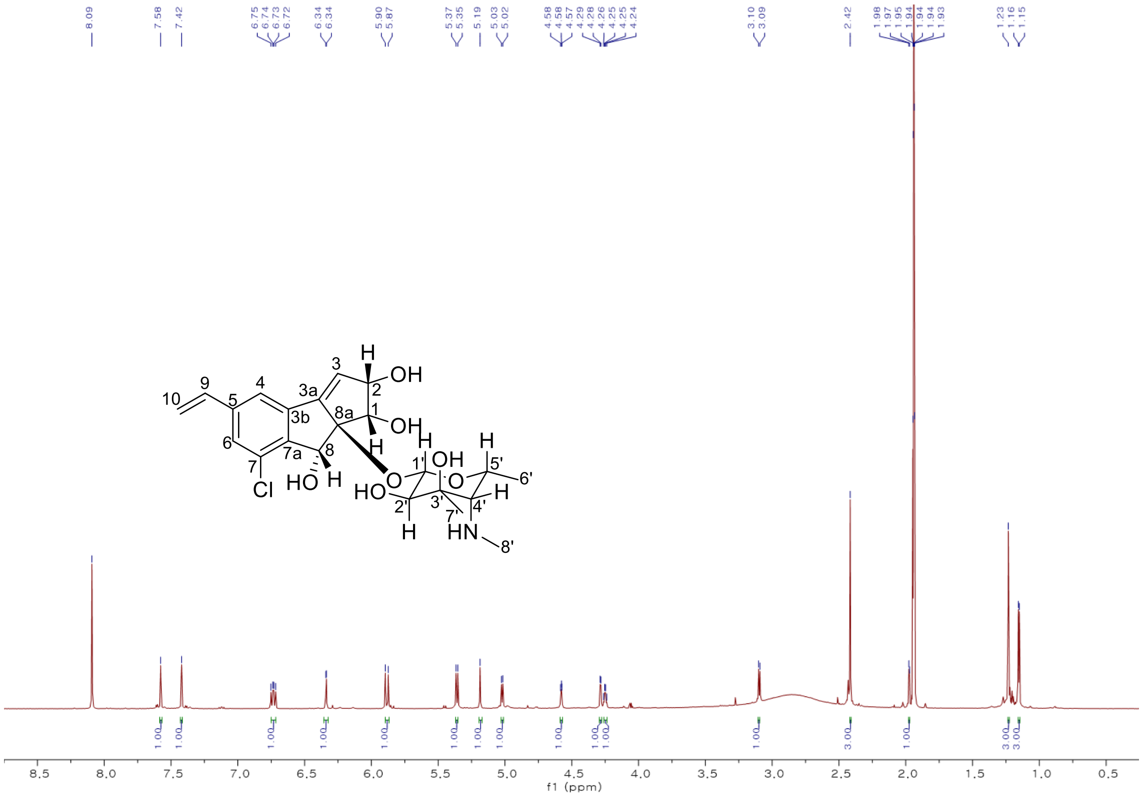

Figure S15. ^{13}C NMR spectrum of jejucarboside D (**3**) at 850 MHz in CD_3CN.

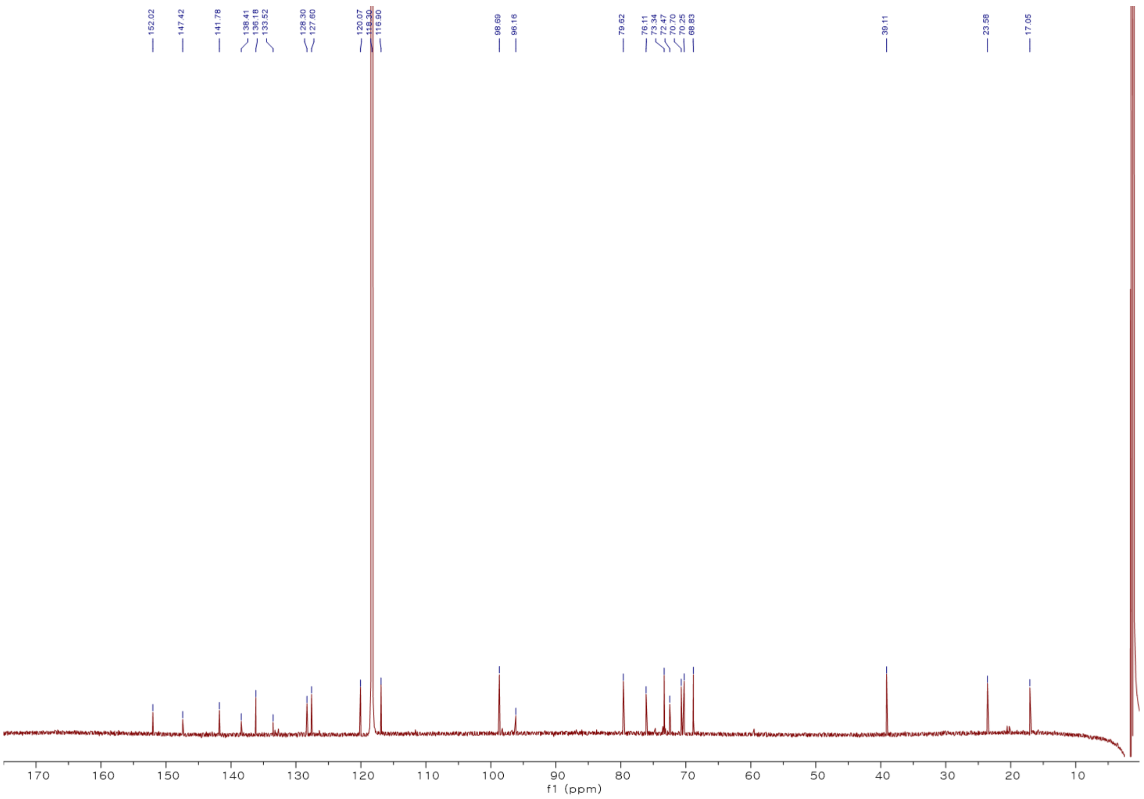

Figure S16. COSY NMR spectrum of jejucarboside D (**3**) at 850 MHz in CD_3CN.

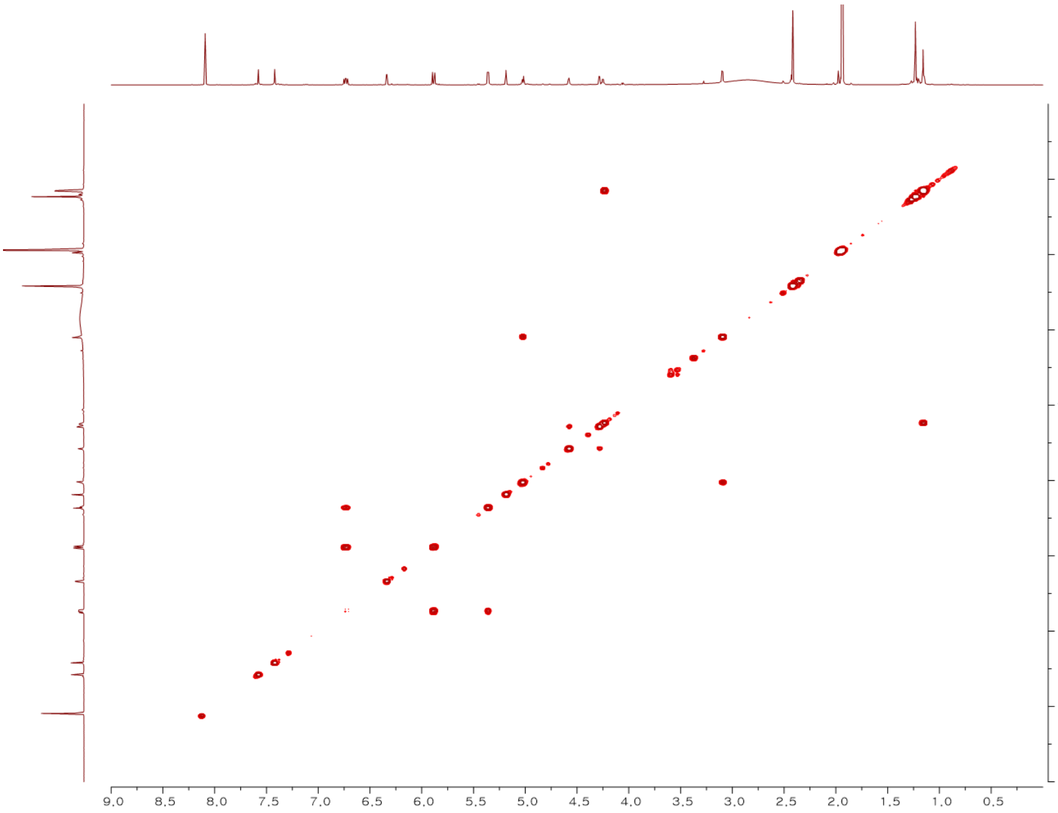

Figure S17. HSQC NMR spectrum of jejucarboside D (**3**) at 850 MHz in CD_3CN.

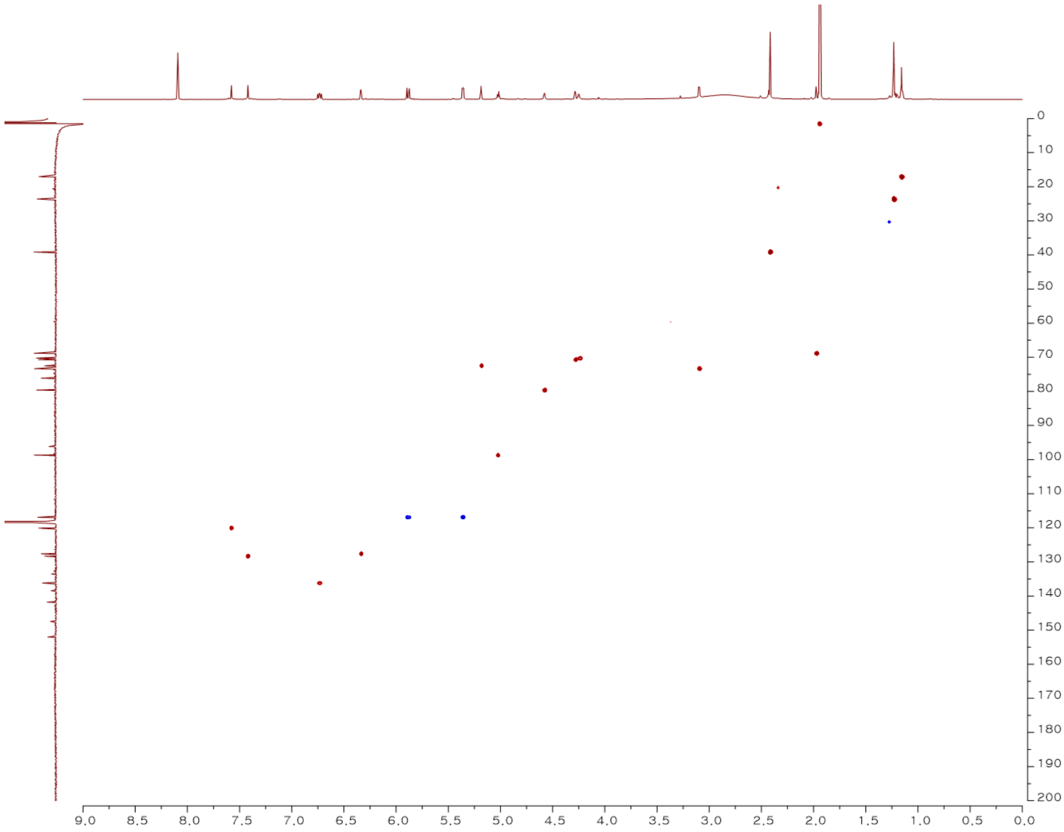

Figure S18. HMBC NMR spectrum of jejucarboside D (**3**) at 850 MHz in CD_3CN.

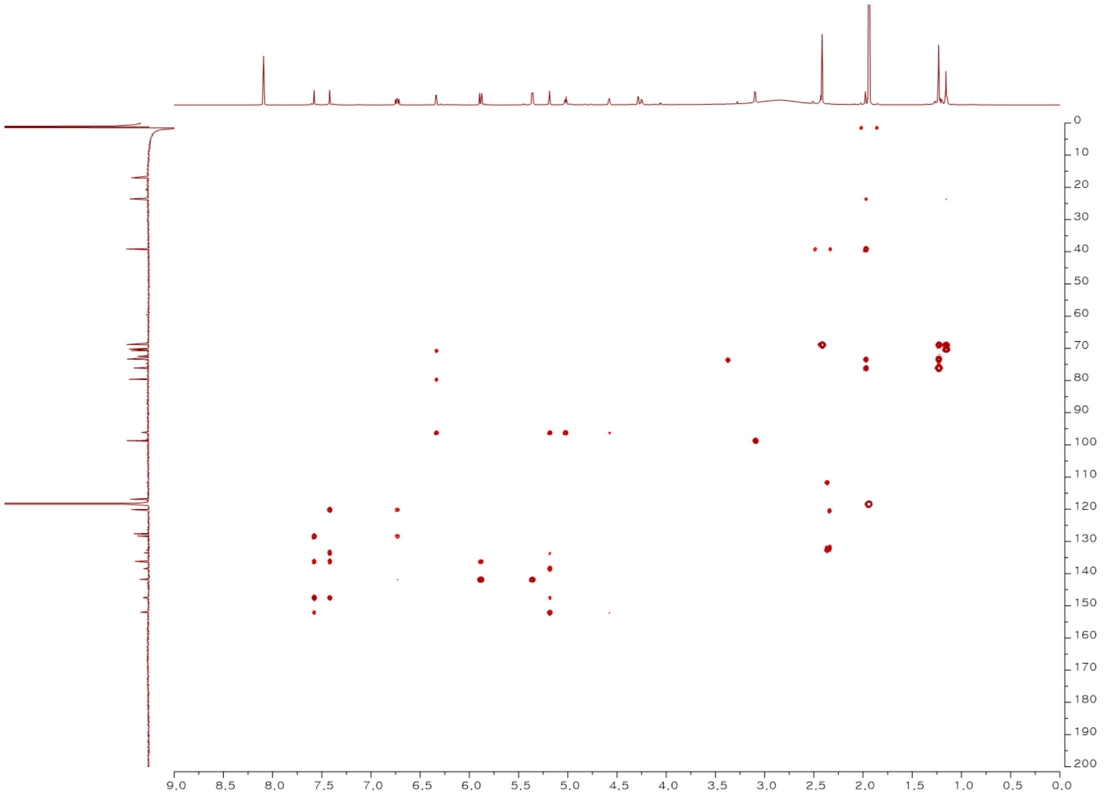

Figure S19. ROESY NMR spectrum of jejucarboside D (**3**) at 850 MHz in CD_3CN.

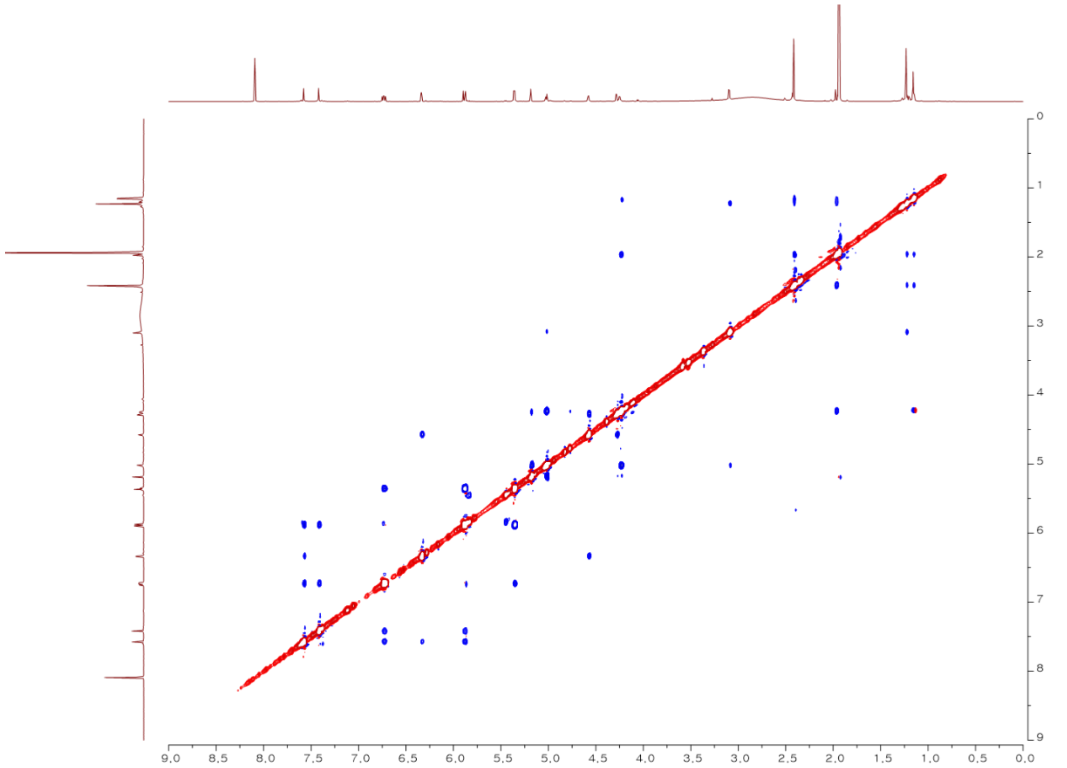

Figure S20. ^{1}H NMR spectrum of jejucarboside E (**4**) at 850 MHz in CD_3CN.

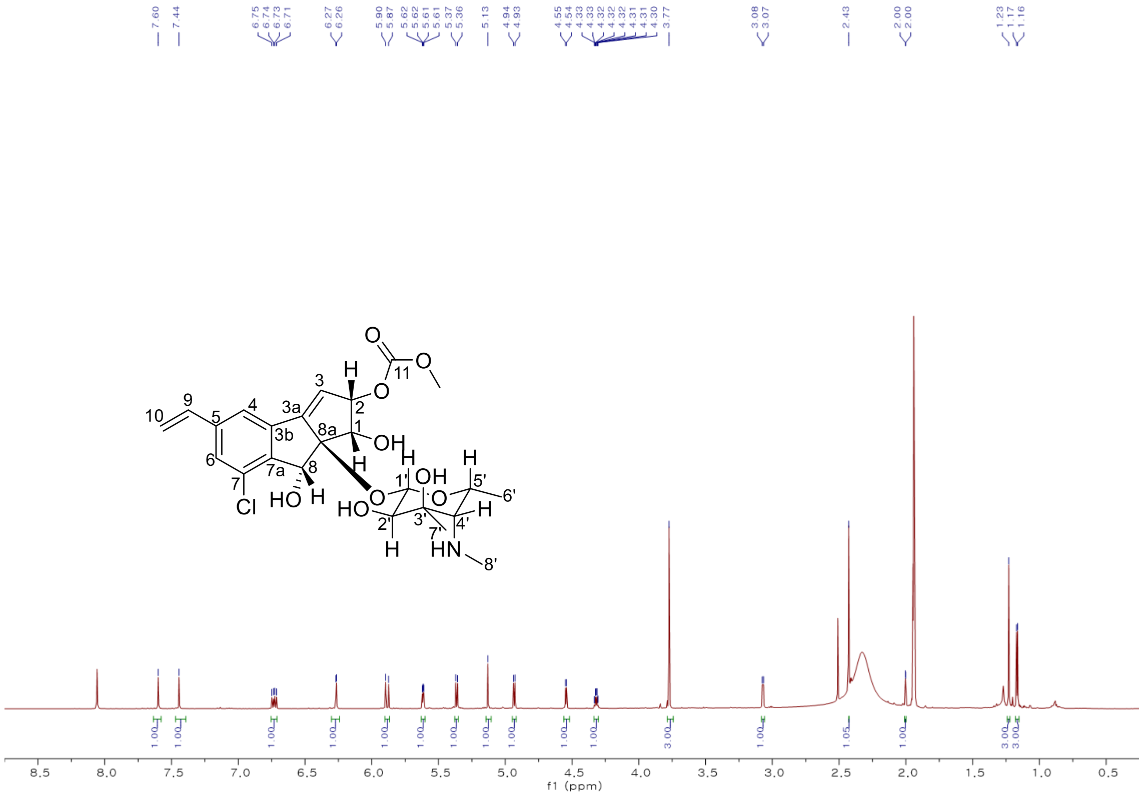

Figure S21. ^{13}C NMR spectrum of jejucarboside E (**4**) at 850 MHz in CD_3CN.

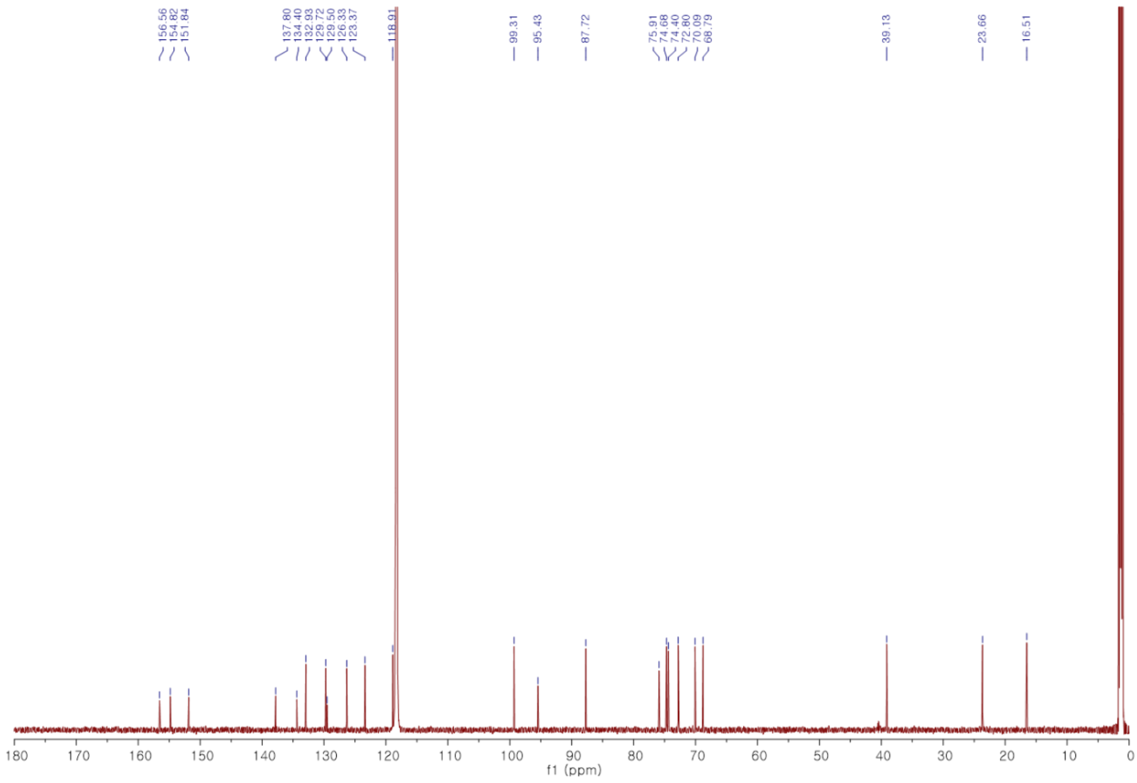

Figure S22. COSY NMR spectrum of jejucarboside E (**4**) at 850 MHz in CD_3CN.

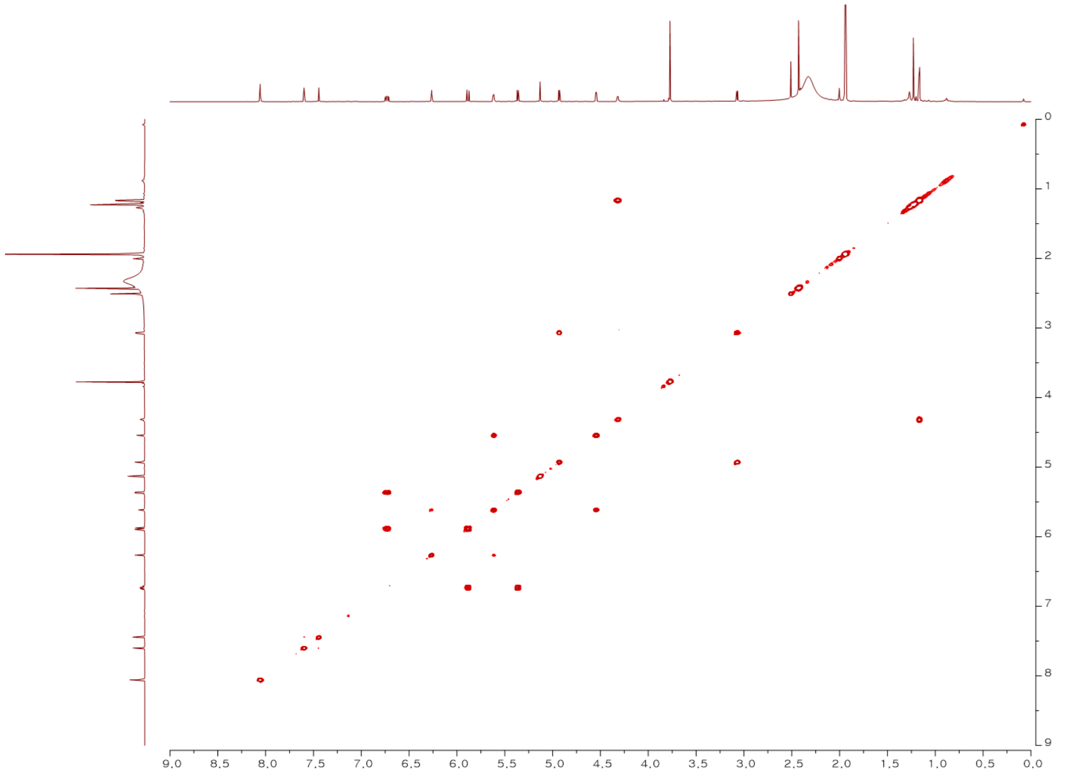

Figure S23. HSQC NMR spectrum of jejucarboside E (**4**) at 850 MHz in CD_3CN.

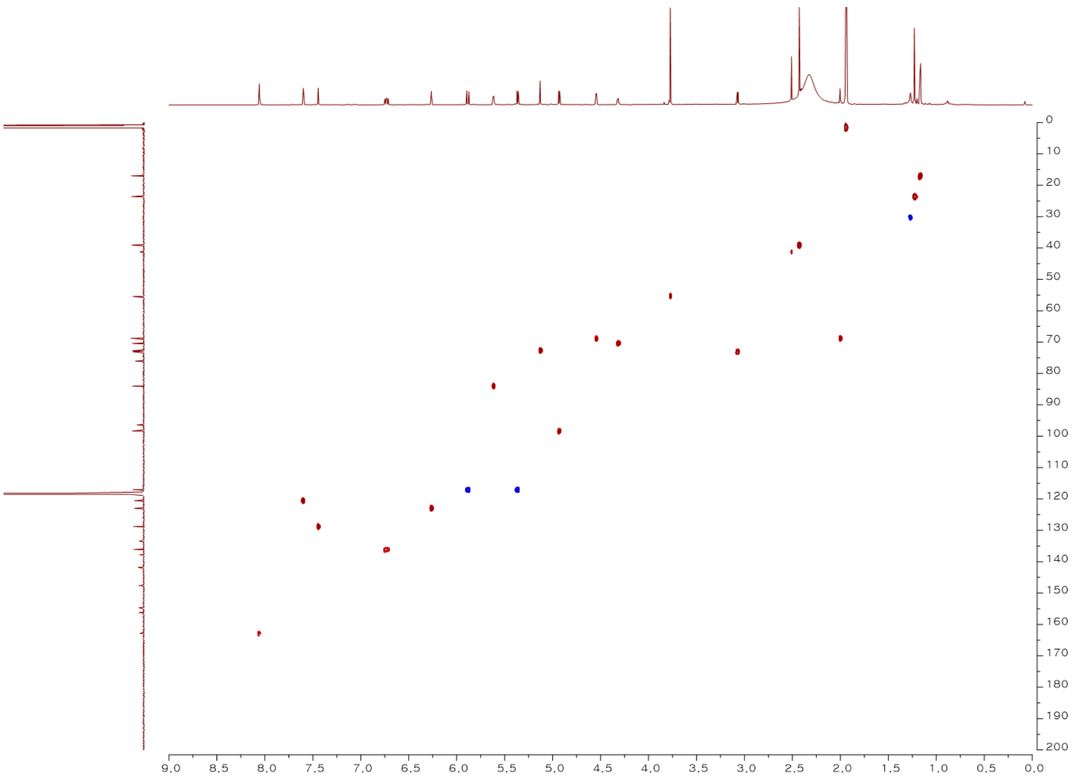

Figure S24. HMBC NMR spectrum of jejucarboside E (**4**) at 850 MHz in CD_3CN.

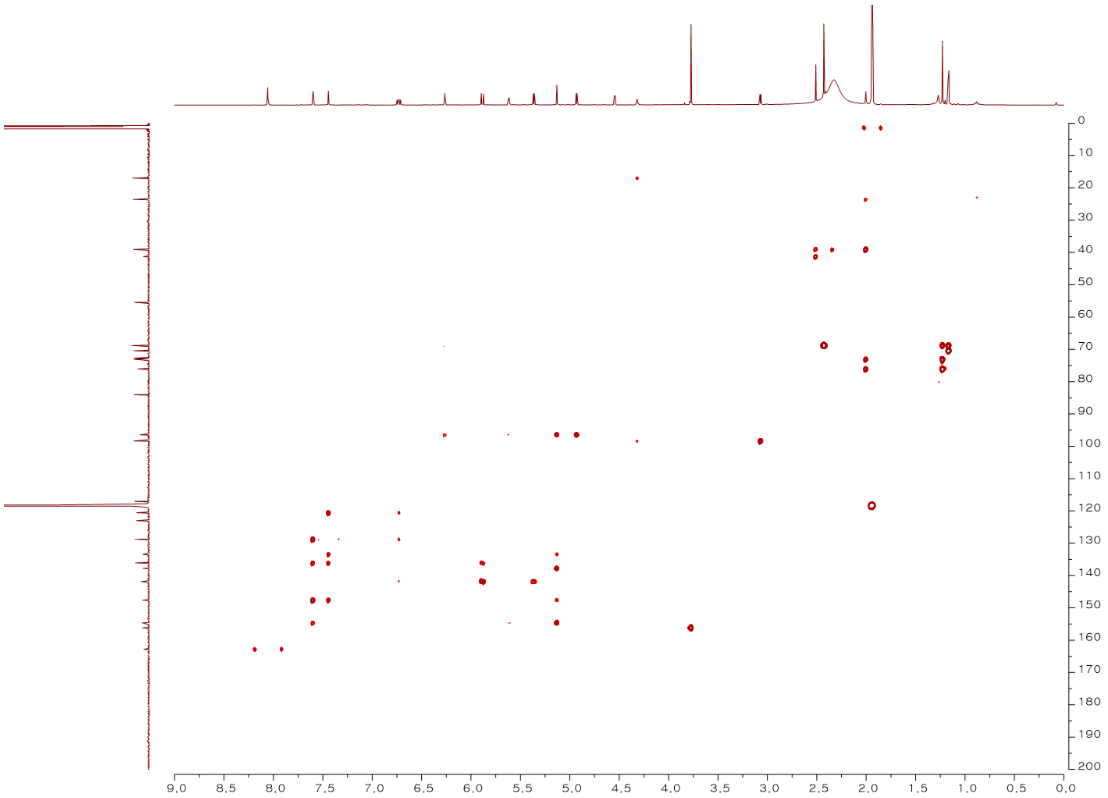

Figure S25. ROESY NMR spectrum of jejucarboside E (**4**) at 850 MHz in CD_3CN.

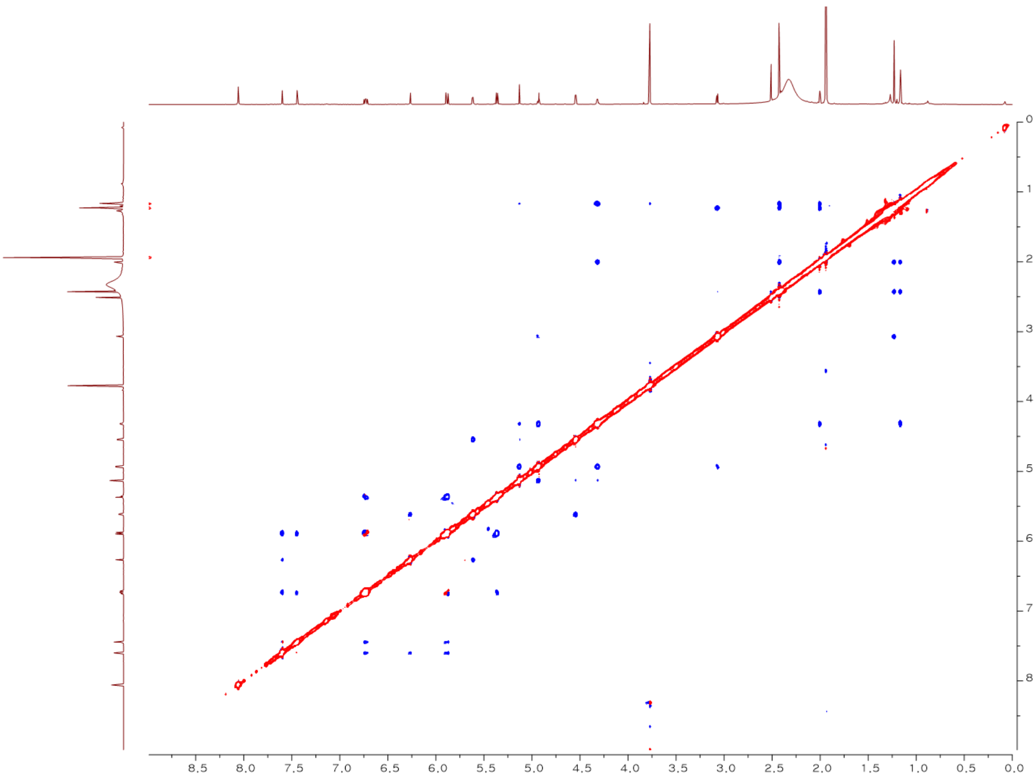

Table S1. NMR spectroscopic data of jejucarboside A (800 MHz, CD_3CN) and jejucarbosides B-E (**1-4**) (850 MHz, CD_3CN).

	**Jejucarbosde A**		**Jejucarboside B (1)**		**Jejucarboside C (2)**		**Jejucarboside D (3)**		**Jejucarboside E (4)**	
**Position**	δ_C, Type	δ_H, Mult (J in Hz)	δ_C, Type	δ_H, Mult (J in Hz)	δ_C, Type	δ_H, Mult (J in Hz)	δ_C, Type	δ_H, Mult (J in Hz)	δ_C, Type	δ_H, Mult (J in Hz)
**1**	74.4, CH	5.32, d (7.0)	74.6, CH	5.34, d (7.0)	70.5, CH	4.25, d (5.5)	70.7, CH	4.28, d (5.0)	68.9, CH	4.54, d (6.0)
**2**	87.7, CH	5.59, dd (7.0, 3.0)	87.5, CH	5.57, dd (7.0, 3.0)	79.8, CH	4.59, dd (5.5, 3.0)	79.6, CH	4.58, dd (5.0, 3.0)	84.0, CH	5.61, dd (6.0, 3.0)
**3**	123.4, CH	6.53, d (3.0)	122.4, CH	6.30, d (3.0)	129.9, CH	6.57. d (3.0)	127.6, CH	6.34, d (3.0)	123.0, CH	6.27, d (3.0)
**3a**	154.8, C		156.5, C		150.3, C		152.0, C		154.6, C	
**3b**	134.4, C		137.7, C		134.7, C		138.4, C		137.8, C	
**4**	129.5, C		120.3, CH	7.68, s	129.6, C		120.1, CH	7.58, s	120.5, CH	7.60, s
**5**	137.8, C		142.1, C		137.5, C		141.8, C		141.8, C	
**6**	129.7, CH	7.73, d (8.0)	129.2, CH	7.49, s	128.8, CH	7.63, d (8.0)	128.3, CH	7.42, s	128.8, CH	7.44, s
**7**	126.3, CH	7.46, d (8.0)	133.2, C		126.5, CH	7.40, d (8.0)	133.5, C		133.5, C	
**7a**	151.8, C		146.6, C		152.6, C		147.4, C		147.6, C	
**8**	74.7, CH	5.01, s	73.3, CH	5.21, s	73.8, CH	5.01, s	72.5, CH	5.19, s	72.7, CH	5.13, s
**8a**	95.4, C		95.1, C		96.6, C		96.2, C		96.4, C	
**9**	132.9, CH	7.16, dd (18.0, 11.0)	135.9, CH	6.76, dd (18.0, 11.0)	133.2, CH	7.13, dd (17.5, 11.0)	136.2, CH	6.75, dd (18.0, 11.0)	136.1, CH	6.73, dd (17.5, 11.0)
**10**	118.9, CH_2	5.52, dd (11.0, 1.0)	117.4, CH_2	5.40, d (11.0)	118.3, CH_2	5.46, d (11.0)	116.9, CH_2	5.36, d (11.0)	117.1, CH_2	5.36, d (11.0)
		5.91, dd (18.0, 1.0)		5.92, d (18.0)		5.85, d (17.5)		5.88, d (18.0)		5.88, d (17.5)
**11**	156.6, C		156.5, C						156.2, C	
**11-OMe**									55.5, CH_3	3.77, s
**1’**	99.3, CH	4.27, d (7.5)	99.7, CH	4.38, d (7.5)	98.6, CH	4.95, d (8.0)	99.3, CH	5.02, d (8.0)	98.3, CH	4.93, d (8.0)
**2’**	72.8, CH	3.01, d (7.5)	72.8, CH	2.99, d (7.5)	73.3, CH	3.08, d (8.0)	72.8, CH	3.09, d (8.0)	73.1, CH	3.07, d (8.0)
**3’**	75.9, C		76.0, C		76.1, C		75.9, C		76.1, C	
**4’**	68.8, CH	1.88, m	68.8, CH	1.88, s	68.8, CH	1.95, d (1.5)	68.8, CH	1.97, d (1.5)	68.8, CH	2.00, d (1.5)
**5’**	70.1, CH	3.86, m	70.0, CH	3.97, q (6.5)	70.3, CH	4.20, qd (6.5, 1.5)	70.1, CH	4.24, qd (6.5, 1.5)	70.4, CH	4.32, qd (6.5, 1.5)
**6’**	16.5, CH_3	1.08, d (6.5)	16.6, CH_3	1.10, d (6.5)	17.0, CH_3	1.13, d (6.5)	16.5, CH_3	1.15, d (6.5)	17.0, CH_3	1.17, d (6.5)
**7’**	23.7, CH_3	1.20, s	23.6, CH_3	1.19, s	23.6, CH_3	1.22, s	23.7, CH_3	1.23, s	23.6, CH_3	1.23, s
**8’**	39.1, CH_3	2.42, s	39.1, CH_3	2.40, s	39.1, CH_3	2.41, s	39.1, CH_3	2.41, s	39.1, CH_3	2.43, s

Table S2. Composition of media used for culture optimization.

**YEME**	**A1C**
Yeast 4g/L<br>Glucose 4g/L<br>Malt 10g/L<br>Sea salt 14g/L	Starch 10g/L<br>Yeast 4g/L<br>Phytone peptone 2g/L<br>Calcium Carbonate 1g/L<br>Sea salt 14g/L
**YEME+Humic acid**	**YEME+L-ornithine**
Yeast 4g/L<br>Glucose 4g/L<br>Malt 10g/L<br>Humic acid 1g/L<br>Sea salt 14g/L	Yeast 4g/L<br>Glucose 4g/L<br>Malt 10g/L<br>L-Ornithine 2g/L<br>Sea salt 14g/L
**YEME+Humic acid+ L-ornithine**	**A1**
Yeast 4g/L<br>Glucose 4g/L<br>Malt 10g/L<br>Humic acid 1g/L<br>L-Ornithine 2g/L<br>Sea salt 14g/L	Starch 10g/L<br>Yeast 4g/L<br>Peptone 2g/L<br>Sea salt 14g/L
**DSY**	**GLY**
Dextrin 10g/L<br>Soytone (soybean meal) 4g/L<br>Yeast 1g/L<br>NaCl 0.5g/L<br>Sea salt 14g/L	Glycerol 20g/L<br>Lactose 10g/L<br>Malt 5g/L<br>Yeast 5g/L<br>Calcium Carbonate 1g/L<br>Sea salt 14g/L
**modified K**	**YPM**
Yeast 3g/L<br>Glucose 2g/L<br>Mannitol 2g/L<br>Malt 5g/L<br>Starch 5g/L<br>Soytone 5g/L<br>Calcium Carbonate 1g/L<br>Sea salt 14g/L	Yeast 2g/L<br>Mannitol 4g/L<br>Peptone 2g/L<br>Sea salt 14g/L

Phylogenetic analysis of Enediyne Core Gene Amplicons

Translated amino acid sequences of the amplicons and references were aligned using MUSCLE (v5.1) [1], and gap-free consensus regions were extracted for their phylogenetic analysis. Phylogenetic analysis of the alignments was proceeded using RAxML (v8.2.11) [2] plugin installed in Geneious Prime software (v2023.0.4) with GAMMA DAYHOFF protein model and 100 bootstrap replicating number. The final bootstrap support percentage values were calculated by best-scoring maximum likelihood tree searching option in the plugin.

Figure S26. Phylogenetic tree of the amplicons.

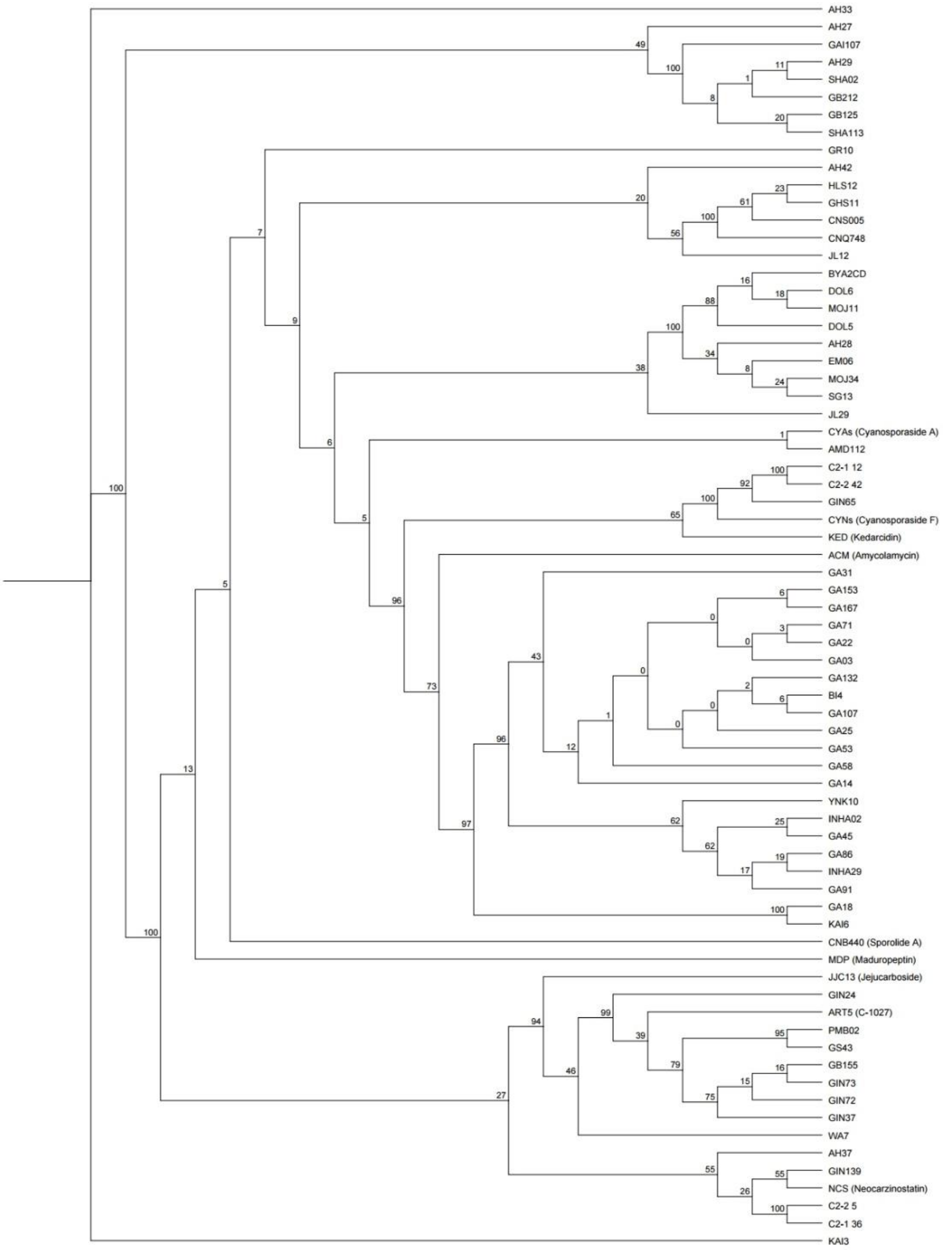

References

(1) Edgar, R. C. MUSCLE: multiple sequence alignment with high accuracy and high throughput. *Nucleic Acids Res.* **2004**, *32*, 1792-1797. DOI: 10.1093/nar/gkh340.

(2) Stamatakis, A. RAxML version 8: a tool for phylogenetic analysis and post-analysis of large phylogenies. *Bioinformatics* **2014**, *30*, 1312-1313. DOI: 10.1093/bioinformatics/btu033.
